# Leukotriene B4 regulates T cell recognition and control of MCMV in mucosal tissues

**DOI:** 10.1016/j.mucimm.2025.08.002

**Published:** 2025-08-18

**Authors:** Lauren E. Springer, Han-Zhi Rao, Oliver Abinader, Ramkrishna Mitra, Christopher M. Snyder

**Affiliations:** a Department of Microbiology and Immunology, Thomas Jefferson University, Philadelphia, PA, USA; b Division of Biostatistics and Bioinformatics, Department of Pharmacology, Physiology and Cancer, Thomas Jefferson University, Philadelphia, PA, USA

**Keywords:** Leukotriene, 5-lipoyxgenase, Inflammation, Cytomegalovirus, T cells, Cytokines

## Abstract

Lipid mediators play important, yet poorly understood roles in regulating immune responses. Cytomegalovirus (CMV) is a herpesvirus that persists in mucosal tissues. Prior work suggests that leukotrienes, a class of inflammatory lipid mediators, contribute to viral control. Infection with murine (M)CMV altered leukotriene and other lipid mediator production in the nasal mucosa, lungs and salivary glands of mice. Mice lacking the receptor for leukotriene B4 (BLT1^−/−^) had increased viral titers at early timepoints in the nasal mucosa and lungs and produced less interferon (IFN)-γ in both tissues, altering the balance between IFN-γ and interleukin (IL)-10. Importantly, viral control in BLT1^−/−^ mice was restored by IL-10 blockade, showing that leukotriene B4 promotes an optimal IFN-γ/IL-10 balance in these mucosal sites during acute infection. BLT1^−/−^ T cells showed no defects in the ability to produce IFN-γ, but their gene expression profiles suggested reduced activation and altered migratory capacity. MCMV-specific T cells compete for access to infected cells. Remarkably, when in competition with wild-type T cells, BLT1^−/−^ T cells competed poorly for antigen, resulting in reduced expansion. These data suggest that leukotriene B4 promotes control of CMV by optimizing T cell encounters with infected targets, maintaining the balance between IFN-γ and IL-10.

## Introduction

Cytomegalovirus (CMV) is a β-herpesvirus that establishes life-long latent/persistent infections in most people throughout the world.^1–2^ CMV causes asymptomatic infections in immunocompetent individuals but can cause morbidity and mortality in immunocompromised and immune immature hosts.^3–5^ In fact, CMV is the most common infectious complication of transplantation and the leading cause of congenital infections worldwide.^6–8^ Immunocompromised individuals frequently present with complications in the gastrointestinal tracts and central nervous system, along with life-threatening complications in the lungs.^9^ In addition, one out of five children born with congenital CMV infection will develop permanent medical complications such as sensorineural hearing loss and developmental disabilities.^10–12^ There are no vaccines available and the factors that dictate immune control of CMV are incompletely understood.

Increasing evidence points to the oral/nasal mucosa (NM) as an initial site of infection,^13–19^ leading to infection of the lungs and airways, and systemic spread of CMV via a leukocyte-associated viremia.^13,20–26^ This enables the virus to reach sites of shedding, including the salivary glands (SG) and kidneys. In these organs, CMV is well-known to persist, and it is shed in saliva and urine for prolonged periods of time, particularly in children.^27–28^ While CMV primarily infects fibroblasts, endothelial cells, epithelial cells, monocytes, and macrophages, the latter are crucial to initiate an early inflammatory cascade during acute infection.^29–30^ Work with the murine (M)CMV model has shown that T cells play a vital role in restricting viral replication and reactivation, with CD8^+^ T cells being effective in most tissues^31–32^ except the SG, where CD4^+^ T cell-derived interferon-gamma (IFN-γ) plays a critical role in viral control.^33–35^ In the lungs, IFN-γ secretion by NK cells, CD4^+^ T cells, and CD8^+^ T cells contribute to local control of MCMV^36^ and we have shown that T cells regulate viral loads in the NM as well during acute infection.^22^

Because CMV leads to lifelong latency and sporadic low-level reactivation, T cell responses to a subset of immunodominant epitopes arise and accumulate over time, becoming a large proportion of the total CD8^+^ T cell pool.^37^ This phenomenon, known as “memory inflation”, begins shortly after acute infection and is characterized by repeated stimulation of T cells by infected targets throughout the body.^38–43^ T cells compete with each other for access to these infected cells, with successful T cell responses and T cell clones becoming dominant, while unsuccessful clones and responses are suppressed.^37,44–46^ These T cell encounters with infected targets effectively “silence” viral transcription and thus, play an important role in controlling the virus.^37,47^

Lipid mediators of inflammation and resolution act as potent signaling molecules to regulate cellular responses to both sterile and pathogenic inflammation.^48–50^ Increasing evidence suggests that these mediators play significant roles in viral infections, including CMV infections. These lipids are derived from essential polyunsaturated fatty acids – arachidonic acid (AA), docosahexaenoic acid (DHA), or eicosapentaenoic acid (EPA); either biosynthesized from precursors or received directly from diet.^51^ Importantly, oxygenation of the omega-6 fatty acid, AA, leads mainly, but not exclusively, to inflammatory lipid mediators, whereas oxygenation of omega-3 fatty acids, EPA or DHA, leads to specialized pro-resolving mediators, which actively promote resolution and clearance of debris.^48–53^ The enzyme 5-lipoxygenase (5-LO) is central to many of these biosynthetic pathways, including the production of key acute bioactive inflammatory mediators – leukotrienes, which are derived from AA (Fig. S1C).^54–56^ There are five known leukotrienes: leukotriene B4 (LTB4) and the cysteinyl leukotrienes LTC4, LTD4, LTE4 and LTF4.^57–61^ These molecules have a broad range of functions including, but not limited to, acting as chemoattractants for innate and adaptive immune cells, inducing inflammatory cytokine production, and inducing mucus hypersecretion and smooth muscle cell hyperplasia.^57–60^ Importantly, the leukotriene pathway is frequently targeted clinically to treat allergies and asthma.^62–67^

Prior work has suggested that CMV induces the production of leukotrienes, which may promote both inflammation and viral control. However, little is known about these processes and their impact on infection. Human (H)CMV infection upregulates 5-LO in various cell types, as well as cyclooxygenase-2 (COX-2), which processes AA into prostaglandins.^68–77^ Moreover, infection of vascular smooth muscle cells led to increased production of LTB4.^71^ LTB4 treatment of infected human peripheral blood leukocytes reduced HCMV titers in a concentration-dependent manner, and the effect was dependent on the LTB4 receptor 1 (BLT1).^78^ Consistent with this in vitro work, mice lacking 5-LO (5-LO^−/−^), and thus unable to make any leukotrienes, showed significantly increased loads of MCMV in the SG, and LTB4 treatment of these mice reduced the SG viral loads.^79^ However, the mechanisms behind this finding remain unknown, and it is still not clear whether the impact is limited to the SG. It is important to note that mice lacking the 5-LO enzyme lack multiple inflammatory lipid mediators as well as several specialized pro-resolving mediators, while substrates of 5-LO can be converted into alternative bioactive lipids by other enzymes,^80–82^ complicating the interpretation of the increased viral loads. An older report suggested that LTB4 may promote IFN-γ production by T cells in culture.^83^ While IFN-γ-expressing T cells are critical for controlling CMV infection,^31^ whether this can account for the impact of LTB4 in MCMV infected mice has not been tested.

In this study, we use the MCMV model to investigate the role of LTB4 signaling on viral control. Our data show that LTB4, via its receptor BLT1, modulates IFN-γ production and control of MCMV in the NM, lungs and SG. We further show that LTB4 promotes T cell access to infected cells and thus modulates the critical balance between IFN-γ and IL-10 in these mucosal sites. Together, our data suggest that LTB4 promotes control of CMV by optimizing T cell encounters with infected targets.

## Materials and methods

### Ethics statement

All animal experiments and procedures were reviewed and approved by the Thomas Jefferson University Institutional Animal Care and Use Committee, which follows the Office of Laboratory Animal Welfare Public Health Service Policy on Humane Care and Use of Laboratory Animals (Animal Welfare Assurance Number: D16–00051). All animals were maintained in the specific-pathogen-free facility of the Thomas Jefferson University and treated in accordance with the Association for Assessment and Accreditation of Laboratory Animal Care (AAALAC) International regulations.

### Mice

Six- to thirteen-week-old mice were used for all experiments. C57BL/6J (B6) mice, B6.129S2-Alox5^tm1Fun/J^ (5-LO^−/−^) mice, B6.SJL-*Ptprc*^*a*^ *Pepc*^*b*^/BoyJ (CD45.1) mice, and B6.129P2-Tcrb^tm1Mom/J^ (TCRβ^−/−^) mice were all purchased from the Jackson Laboratory. The BLT1^−/−^ mice on the B6 background were described previously^84^ and kindly provided by Dr. Haribabu Bodduluri (University of Louisville). The LTC4S^−/−^ mice on the B6 background were described previously^85^ and kindly provided by Dr. Seth Furgeson (University of Colorado). All mouse strains were maintained in our animal colony.

### Viruses

Murine cytomegalovirus strain K181 was used for all experiments. Virus was propagated and titrated by plaque assay on M2–10B4 cells as previously described.^86^

### Experimental infection

Mice under isoflurane anesthesia were infected intranasally (i.n.) with 10^6^ pfu MCMV-K181 in 20 μL phosphate-buffered saline (PBS) (10 μL per naris) as previously described^22,87^ for all experiments, except for mass spectrometry data, in which mice were i.n. infected with 10^6^ pfu MCMV-K181 in 50 μL PBS (25 μL per naris).

### Mucosal organ virus titration

Dissections and virus titration from the nasal mucosa (NM), lungs, and salivary glands (SG) were performed as described previously,^22,87^ with the following changes. Twenty-five percent homogenates [weight/volume (w/v)] of tissue were individually prepared from NM, lungs, and SG for virus titrations. Tissues were weighed and homogenized in 1.5 mL tubes using a pestle with a small amount of sterile sand prior to suspension in complete media [RPMI-1640 with L-glutamine (Corning) supplemented with 10 % fetal bovine serum (FBS) and 1% penicillin–streptomycin (PS)]. Homogenates were centrifuged (3,000 × g for 20 min) and supernatants were collected for plaque assay. Plaque assays were performed on M2–10B4 cells (ATCC) as previously described,^22,86^ with minor modifications. Specifically, the M2–10B4 cells were ~ 80%–100% confluent at the start of the assay, and NM, lungs, and SG were serially diluted in 3-fold increments. All tissue homogenates were removed by vacuum suction after 1.5 h incubation, and wells containing SG homogenates were rinsed with 1 mL of PBS. After rinsing, 1 mL of fresh media was added to each well, which were then covered with 4 mL of carboxymethyl cellulose (CMC) diluted in complete media.

### Lipid quantification MS-MS

NM, lungs, and SG at 0, 1-, 3-, 7-, and 14-days post infection (DPI) were collected from B6 mice into 1.5 mL tubes and flash frozen in liquid nitrogen. Lipid analysis was performed at the University of California San Diego (UCSD) Lipidomics Core.^88^

### Cell culture and primary cell isolation and culture

The M2–10B4 cell line was cultured in complete media at 37 °C with 5 % carbon dioxide. The bone marrow from femurs and tibias of B6 mice and 5-LO^−/−^ mice were used to prepare bone marrow macrophages. Nonpolarized bone marrow derived macrophages were prepared and cultured as previously described.^89^ The ears and tail of B6 mice and 5-LO^−/−^ mice were used to prepare fibroblasts as described.^90^

### Multi-step growth curves in vitro

For growth of MCMV in the absence of 5-LO or in the presence of leukotrienes, 2.5 × 10^5^ bone marrow derived macrophages or ex vivo fibroblasts were seeded into 6-well plates. One day later, cells were pre-treated with leukotriene (LT) mixture including 10 nM each of LTB4, LTC4, LTD4, LTE4, LTF4, and N-acetyl LTE4 (Cayman Chemicals) or vehicle alone for 2.5 h before infecting with MCMV-K181 at a multiplicity of infection (MOI) of 0.1 for 1.5 h without centrifugal enhancement. Lipids stored in ethanol were diluted in PBS and vehicle was prepared as ethanol in PBS. After 1.5 h, the supernatant was collected for input virus titer (labeled as day −1), and cells were washed with PBS and supplemented with 2 mL of fresh complete media. Lipid mixture and vehicle were added to wells daily without changing media (25 μL as to not significantly alter the volume). Cells and media were scraped and collected from duplicate wells immediately after the wash (day 0) and on days 1, 3, 5 and 7 after infection.

### ELISAs

Collected NM, lungs, and SG were suspended in complete media at 50 % w/v (NM) or 25 % w/v (lungs and SG) and dissociated in a 1.5 mL tube with three to four 3.0 mm TriplePure M Bio grade high impact zirconium beads (Benchmark Scientific) using a mini bead beater (Bio-spec Products) for 30 s. Homogenates were centrifuged (3,000 ×g for 10 min) and supernatants were collected for ELISA. ELISA MAX Deluxe mouse sets were used for IFN-γ and IL-10 ELISA cytokine assays (Biolegend) performed following the manufacturer’s directions on supernatant from organs. Plates were read using Synergy 2 plate reader (Biotek) at 450 nm.

### In vivo IL-10R blockade

To block IL-10 signaling, mice were injected i.p. with 250 μg anti-CD210 (anti–IL-10R clone 1B1.3A; BioxCell) or rat IgG1 as a control (clone HRPN; BioxCell) at 1 and 7 DPI as previously described.^87^

### Mucosal organ dissociation for flow cytometry

NM, lungs, and SG were collected into 10 mL of T cell media [complete media, as described above with the addition of 5.5×10^−5^ M beta-mercaptoethanol (Gibco)]. Spleens (SPs) were collected in 2 mL of media and homogenized by using the back of a syringe against a 70 μM filter. Cells were washed twice with 20 mL of T cell media into a 50 mL conical by use of centrifugation at 400 ×g for 7 min, and resuspended and counted for use in staining. Collected NM, lungs, and SG and were dissociated with the gentleMACS dissociator (Miltenyil Biotec) on lung_01 setting using the C-tubes. Homogenates were centrifuged at 300 ×g for 6 min. Supernatant was removed and 10 mL of digestion media containing 2 mg/mL collagenase type IV (ThermoFisher Scientific) in T cell media were added to each homogenate and vortexed. SG were incubated shaking at 37 °C for 1 h in digestion media. NM and lungs were incubated in same manner but for 45 min. Organs were dissociated a second time with the gentleMACS dissociator on lung_02 setting. Recovered cells were washed twice, counted and stained for flow cytometry (see below). For tetramer staining of antigen-specific T cells and intracellular staining following ex vivo stimulation, cells recovered from the second dissociation were further isolated through Percoll. Percoll dilutions were performed in complete media. Post centrifugation, NM, lung, and SG cell pellets were resuspended in 40 % Percoll (Cytiva). For the SG, tubes were layered with 75 % Percoll, followed by 55 % Percoll, and lastly SG cells resuspended in 40 % Percoll at the top layer. No other layers were used for NM and lung cell separation. Cells were centrifuged for 25 min at 4 °C at 600 × g with low acceleration and deacceleration. Lymphocytes from the NM and lung were collected from the pellet while lymphocytes from the SG were collected from between the 75 and 55 % Percoll layers. Cells were then washed twice with 20 mL of T cell media, resuspended and counted for use in staining.

### Flow cytometry: Intravenous antibodies injection, antibodies, tetramer staining, and FACS analysis

All antibodies were received from Biolegend unless specified otherwise. To distinguish cells localized to the vasculature and parenchyma, mice were intravenously (i.v.) injected with 1.5 μg fluorescently labelled antibodies specific for CD45.2 (PE; clone 104) or panCD45 (BV421, clone 30-F11; eBioscience) 3 min before sacrifice for both innate myeloid and T cell panels. Animals were not perfused, and cells were extracted from tissue as described above. For the innate myeloid cell panel, cells were washed in FACS buffer (DPBS [Cellgro], 1 % FBS, 0.05 % sodium azide) and stained with Fc block (clone 93) for 15 min at 4 °C. Cells were then washed with PBS and stained with Zombie Aqua fixable viability stain (Biolegend) for 15 min at 4 °C, following the manufacturer’s recommended protocol. Cells were then stained in a 50 μL volume for 30 min to 1 h with the following additional antibodies: CD45.2 (APC; clone 104), Ly6G (PEcy5; clone 1AB-Ly6g; eBioscience), CD11b (BV650; clone M1/70), CD11c (APC/Cy7; clone N418), CD24 (BV605; M1/69), CD64 (PE/Cy7 clone X54–5/7.1); MHCII (BV785; clone M5/114.15.2), FcεRI (AF700; clone MAR-1), CD117 (FITC; ACK2) and NK1.1(Percpcy5.5; PK136). For the T cell and tetramer panel, cells recovered from the Percoll fractionation (see above) were first stained with MHC-I tetramers. Tetramers were produced at the NIH tetramer core facility (https://tetramer.yerkes.emory.edu) and loaded with the M45 (HGIRNASFI, D^b^-BV421) or M38 (SSPPMFRV, K^b^-Alex488) peptides. Tetramers were incubated with cells for 20 min at room temperature. Cells were then washed with PBS and stained as above with the Zombie Aqua fixable viability stain and the following antibodies: CD45.1 (APC; clone A20) or CD45.2 (APC; clone 104), CD3 (BUV395; clone 17A2); BD Biosciences), NK1.1 (BUV805; clone PK136; Ebioscience), CD4 (APC/Cy7; clone RM4–5), CD25 (BV711; clone PC61), FOXP3 (AF700; clone MF-14), CD8 (PE/Cy5; clone 53–6.7). In both panels, the cells were washed, fixed, and resuspended in 250 μL of FACS buffer for analysis and analyzed on a BD LSRII or BD FACSymphony A5, and the FCS files were analyzed using FlowJo X (FlowJo, LLC).

### Single cell RNA seq organ processing, cell labelling and sorting

Cells for single cell RNA sequencing groups were pooled from four mice per group. For cell labeling, mice were i.v. injected with CD45.2 (PE) as described above (Flow cytometry: Intravenous antibodies injection, antibodies, tetramer staining, and FACS analysis). Cells from NM, lung, and SG were collected/dissociated as indicated above (mucosal organ dissociation for flow cytometry). After isolation, cells from the four mice were pooled and stained for sorting. All washes and stains were performed in PBS supplemented with 1 % FBS, except for cell viability stain in PBS. Cells were stained with Fc block (clone 93) for 15 min at 4 °C. Then, cells were stained with zombie aqua fixable viability stain for 15 min at 4 °C. NM and lung cells were then stained in 100 μL volume per 5 million cells for 30 min to 1 h with the following additional antibodies: CD45.2 (PE), Siglec-F (PE/Dazzle 594; S17007L), CD19 (PE/Dazzle594; clone 6D5), and FcεRIα (PE/Dazzle594; clone MAR-1). The following TotalSeq antibodies (Biolegend) were used: CD103 (clone 2E7), CD11b (clone M1/70), CD11c (clone N418), CD207 (clone 4C7), CD27 (clone LG.3A10), CD64 (clone X54–5/7.1), CD80 (clone 16–10A1), CD86 (clone GL-1), CD24 (clone M1/69), F4/80 (clone BM8), Ly-6C (clone HK1.4), Ly-6G (1A8), I-A/I-E (clone M5/114.15.2), NK-1.1 (clone PK136), CD335 (clone 29A1.4), SiglecH (clone 551), CD169 (clone3D6.112), CD8a (53–6.7), CD4 (RM4–5), and Rat IgG2a, κ Isotype Ctrl (clone RTK2758). Target cells were positively sorted as live, IV + cells, and cells expressing Siglec-F, CD19, and FcεRIα were excluded. Cells were sorted into RPMI supplemented with 20 % FBS using sheath fluid that was free of EDTA and magnesium as not to affect library preparation.

### Single cell RNA seq library preparation

Chromium Single Cell 3′ Expression Dual Index Library and Chromium Single Cell 3′ Cell Surface Protein Dual Index Library were constructed using the Chromium Next GEM Single Cell 3′ Reagent Kits v3.1 (Dual Index) protocol (10X Genomics).

### Cite-seq data analysis

CITE-seq reads were processed with 10X Genomics cellranger (version 8.0.1) tool. The mouse reference genome mm10 was used to map RNA reads, and known barcodes were used to map the reads from the antibody derived tags (ADTs). We employed the R package Seurat (version 5.1.0)^91^ to preprocess the data including quality control, cell and feature selection. Cells were removed if more than 5 % of UMIs were from mitochondrial genes. Cells with less than 200 genes (nFeature_RNA) or more than 6000 genes were also removed. We retained cells with RNA and protein library sizes not exceeding 50,000 UMIs (nCount_RNA) and 10,000 UMIs (nCount_ADT), respectively. After filtering, the RNA and ADTs were normalized using Seurat’s scTransform^92^ and centralized log ratio method, respectively. The RNA and ADT expression profiles were separately integrated using the function IntegrateData. To learn the relative utility of RNA and protein level in each cell, we employed weighted-nearest neighbor method.^91^ For cell type annotation, we used SingleR^93^ and ImmGen reference transcriptome available in the R Bioconductor package celldex, which comprises microarray profiles of pure mouse immune cells. This defined T cells/NK cells/NK-T cells, Innate Lymphoid Cells (ILCs), γδ T cells, neutrophils, monocytes, macrophages, dendritic cells, and a small number of B cells, endothelial cells, fibroblasts and mast cells. Cellular clusters were further analyzed with the loupe browser and cell types were refined with well-known markers as follows: CD4 or CD8 T cells (CD3e^+^, CD3d^+^, CD19^−^, Igkc^low^, Iglc1^low^, Iglc2^low^, J-chain^low^, Zbtb16^−^, Trbc1^+^ or Trbc2^+^ and either CD4^+^/CD8a^−^/CD8b1^−^, or CD4^−^/CD8a^+^/CD8b1^+^; NK cells (Ncr1^+^, Gzmb^+^); ILC1s [Ncr1^+^ (NM) or Ncr1^−^ (Lung), Zbtb16^+^, Itga1 (CD49a)^+^, Itgb3 (CD61)^+^, Tbx21 (Tbet)^+^, Eomes^−^, CD4/8^−^, Trdc^−^]; ILC2 (Gata3^+^, IL5^+^ among ILCs); ILC3 (Rorc^+^, IL17a^+^ among ILCs); γδ T cells (Trdc^+^, CD3e^+^, CD3d^+^); monocyte/macrophages [CD14^+^ or Fcgr3 (CD16)^+^ or Adgre1 (F4/80)^+^ or Fcgr1 (CD64)^+^]. Clusters enriched in wild-type (WT) or BLT1^−/−^ T cells were compared for differential gene expression. Lists of genes upregulated (adjusted p-values < 0.2) in clusters enriched in WT T cells compared to clusters enriched in BLT1^−/−^ T cells were analyzed for pathway overrepresentation using Panther^94^ and Reactome^95^ resources via the Gene Ontology Resource.^96–97^ The scRNA-seq data is available in the Gene Expression Omnibus (GEO) database under GSE306035.

### Mixed bone marrow chimera preparation

To prepare bone marrow chimeras, recipient TCRβ^−/−^ mice were irradiated with 1200 rads using a split dosing schedule of 600 rad followed by a second dose of 600 rad 4 h later. Bone marrow cells from 1 to 2-month-old donor CD45.1 or BLT1^−/−^ mice were collected and red blood cells were lysed using ACK lysis buffer (ThermoFisher Scientific). Remaining cells were washed and resuspended in 100 × 10^6^ cells/ml of complete media. Irradiated mice were reconstituted by intravenous inoculation of 200 μL of mixed bone-marrow cells from the different donors in a 2.5:1 ratio (BLT1^−/−^:CD45.1). Chimeras were rested for six to eight weeks after reconstitution before being used in experiments.

### Peptide stimulation and intracellular cytokine staining

Fourteen days after i.n. infection, NM, lungs, SG and spleens from mixed bone marrow chimera mice were collected as indicated above (mucosal organ dissociation for flow cytometry) for peptide stimulation and subsequent intracellular staining. Stimulation with either cell stimulation cocktail containing phorbol 12-myristate 13-acetate (PMA) and ionomycin (eBioscience), or M45 and M38 peptides followed by intracellular cytokine staining was done as previously described,^39^ with the following minor change of using the Protein Transport Inhibitor Cocktail (Brefeldin A + monensin) (eBioscience) for blocking of the intracellular protein transport process. Cells were chilled on ice until fixed, and stained as described above (Flow cytometry: Intravenous antibodies injection, antibodies, tetramer staining, and FACS analysis) with the Zombie Aqua fixable viability stain and the following surface antibodies: panCD45 (BUV805;clone 30-F11; eBioscience), CD45.1 (APC; clone A20) or CD45.2 (APC; clone 104), CD8 (BUV395; clone 53–6.7; BD Biosciences), CD4 (AF700; clone RM4–5), CD107a (APC/Cy7; clone 1D4B), KLRG1 (BV711; clone 2F1/KLRG1), and CD27 (BV785; clone LG.3A10). Intracellular staining was performed after surface staining using BD Cytofix/Cytoperm Fixation/Permeabilization Kit (BD Biosciences) following manufacturer’s instructions with the following antibodies: CD69 (BV605; clone H1.2F3), FITC (IFN-γ; clone XMG1.2) and IL-10 (BV421; clone JES5–16E3). CD69 was included with intracellular stains as Brefeldin A has been reported to completely block extracellular CD69 expression on mice splenocytes after in vitro stimulation with PMA/ionomycin.^98^ Cells were resuspended in 250 μL of FACS buffer for analysis and analyzed on a BD FACSymphony A5 or A3, and the FCS files were analyzed using FlowJo X (FlowJo, LLC).

### Quantitative PCR

Fourteen days after i.n. infection, mixed bone marrow chimeric mice were i.v. labeled with panCD45 (BV421) as described above (Flow cytometry: Intravenous antibodies injection, antibodies, tetramer staining, and FACS analysis) and cells from recipient NM, lung, and SG were collected as indicated above (mucosal organ dissociation for flow cytometry). Cells were stained for expression of CD45.1 (APC), CD45.2 (PE), CD4 (FITC, clone RM4–5), and CD8 (BV650, clone 52–6.7) and sorted into panCD45 i.v.-negative (parenchyma) wildtype (CD45.1) or BLT1^−/−^ (CD45.2) CD4^+^ and CD8^+^ populations. For sorting, cells were resuspended in 400 μL of PBS supplemented with 1 mM EDTA and 10 % FBS and were collected in 500 μL of RNA later. RNA from all samples was extracted using the RNeasy Mini Kit (QIAGEN) and cDNA was produced using a high-capacity cDNA reverse transcription kit (Applied Biosystems). Transcripts were detected using iTaq Universal SYBR Green (Bio-Rad) with the following primers: IFN-γ: TGAACGCTACACACTGCATCTTGG and CGACTCCTTTTCCGCTTCCTGAG; IL-10: CCCTGGGTGAGAAGCTGAAG and CACTGCCTTGCTCTTATTTTCACA; and RPLP0: GGACCCGAGAAGACCTCCTT and GCACATCACTCAGAATTTCAATGG. All samples were run on the Applied Biosystems StepOne Plus system. Ct values for cytokine transcripts were normalized to Ct values for the housekeeping gene RPLP0 to determine the ΔCt. Data are expressed as 2^−(ΔCt)^.

### Statistics

All data analyses were performed in GraphPad Prism (Version 10.1.1) unless specified otherwise. Statistical tests performed are described in figure legends. Error bars represented standard error of the mean (SEM) unless specified otherwise.

## Results

### The 5-LO pathway and BLT1 signaling promote efficient control of MCMV

**Fig. 1.**
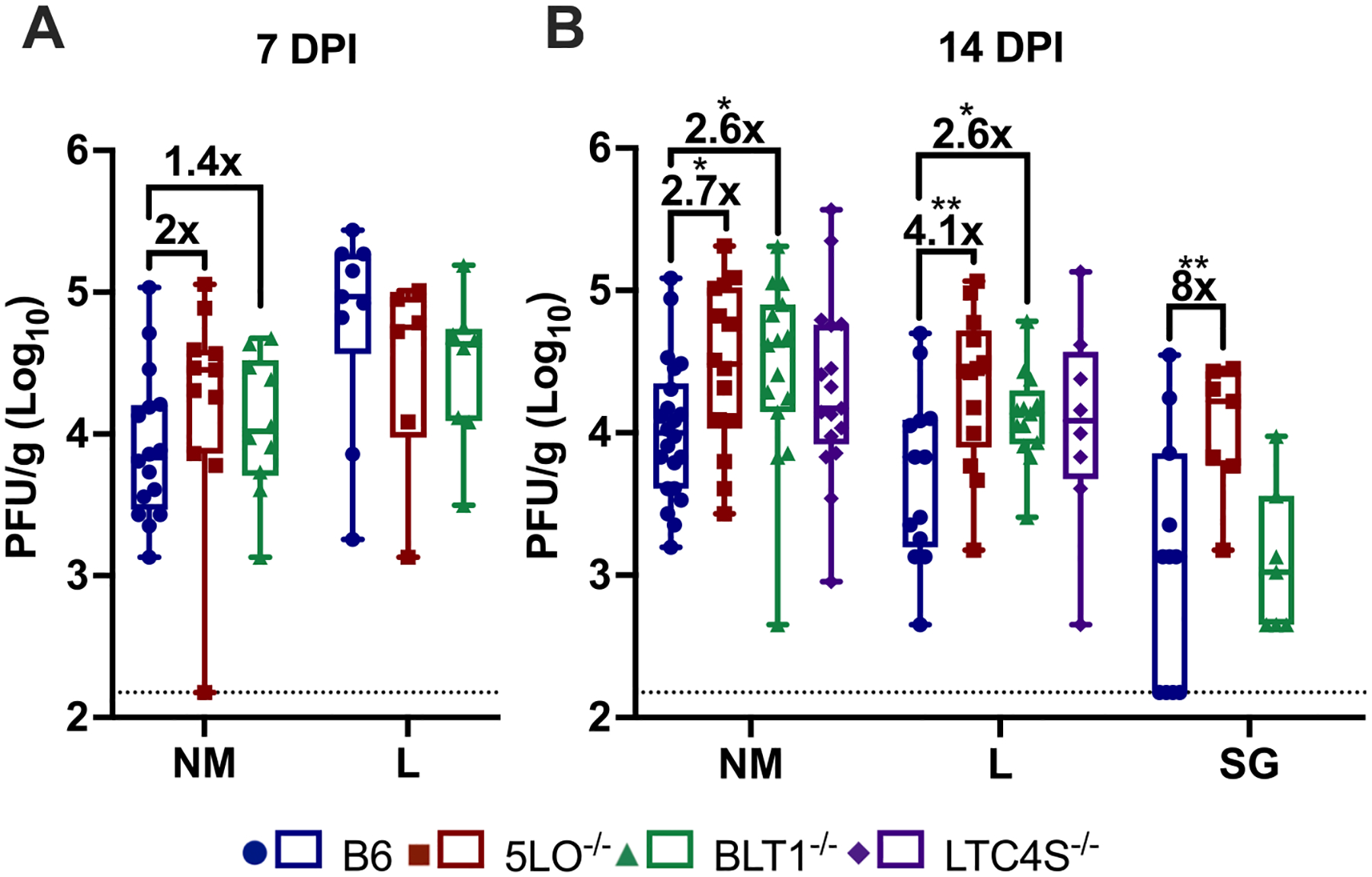
LTB4 promotes viral control in mucosal tissues. Plaque assay quantification of viral titers at (A) 7 DPI and (B) 14 DPI in the NM lungs, and SG of i.n. infected B6, 5LO^−/−^, BLT1^−/−^ and LTC4S^−/−^ mice. Organs collected at various time points were quantified by viral plaque assay. Box and whiskers plots graphed with min. to max., showing individual values of each mouse ranging from n = 7–22 mice per group from at least two independent experiments. Dotted lines represent the assay limit of detection. Fold increases in viral titer calculated by dividing geometric mean of viral titer of knockout mouse group by geometric mean of viral titer of wildtype mice. (Brown-Forsythe and Welch One-way ANOVA of log transformed data) * = p ≤ 0.05, ** = p ≤ 0.01, *** = p ≤ 0.001.

Previous studies indicate a role for 5-LO and LTB4 in CMV control in the SG.^79^ We were interested to test whether 5-LO and/or LTB4 also influenced viral control at mucosal sites of entry. Thus, wild-type C57BL/6J (WT), 5-LO^−/−^, and BLT1^−/−^ mice were infected with K181 strain MCMV via the intranasal (i.n.) route and viral titers were measured (Fig. 1). In the NM and lungs, primary sites of viral entry after i.n. infection, no significant differences in viral loads were detected at 7 days post infection (DPI), although there was a trend towards increasing viral loads in the NM (Fig. 1A). In WT mice, viral titers in the NM persisted between 7 DPI and 14 DPI as expected, while titers in the lungs declined precipitously, which can be traced to T cell responses.^22^ In contrast, in mice lacking either the 5-LO enzyme or the BLT1 receptor, the viral titers were significantly higher by 14 DPI in both the NM and lungs (Fig. 1C). Notably, there was minimal control of viral loads in the lungs between 7 DPI and 14 DPI in 5-LO^−/−^ or BLT1^−/−^ mice (Fig. 1A&B). Loss of the LTC4 synthase (LTC4S), which produces the cysteinyl leukotrienes, resulted in a moderate effect on titer at 14 DPI, which did not reach significance (Fig. 1B). Interestingly, deficiency in the 5-LO enzyme resulted in increased viral titers in the SG at 14 days post infection (Fig. 1B), which is consistent with previous work.^79^ However, this was not replicated by the loss of the BLT1 receptor (Fig. 1B). Thus, viral loads in the SG are not regulated in the same way as in the NM and lungs. Mice lacking the 5-LO enzyme will lack all leukotrienes (Fig. S1C), and both the 5-LO^−/−^ and LTC4S^−/−^ mice produce intermediate lipid metabolites that may have biological effects or be shunted to alternate routes of metabolism resulting in increased amounts of other lipids (e.g. metabolism of AA shunted to COX-2 pathway leading to increased PGE2).^80–82^ Thus, the 5-LO^−/−^ and LTC4S^−/−^ mouse models have an added complexity, whereas BLT1-deficient mice produce conventional amounts of lipids but solely lack signaling through the BLT1 receptor. Regardless, the elevated viral loads in the NM and lungs were transient. By 35 and 63 DPI, viral loads were comparable in 5-LO^−/−^ and BLT1^−/−^ mice (Fig. S1A–B). Together, these results suggest that 5-LO metabolites modulate acute MCMV infection, and that LTB4 signaling specifically promotes viral control at mucosal sites of viral entry.

### MCMV infection induces changes in the patterns of lipid mediator production in mucosal tissues

**Fig. 2.**
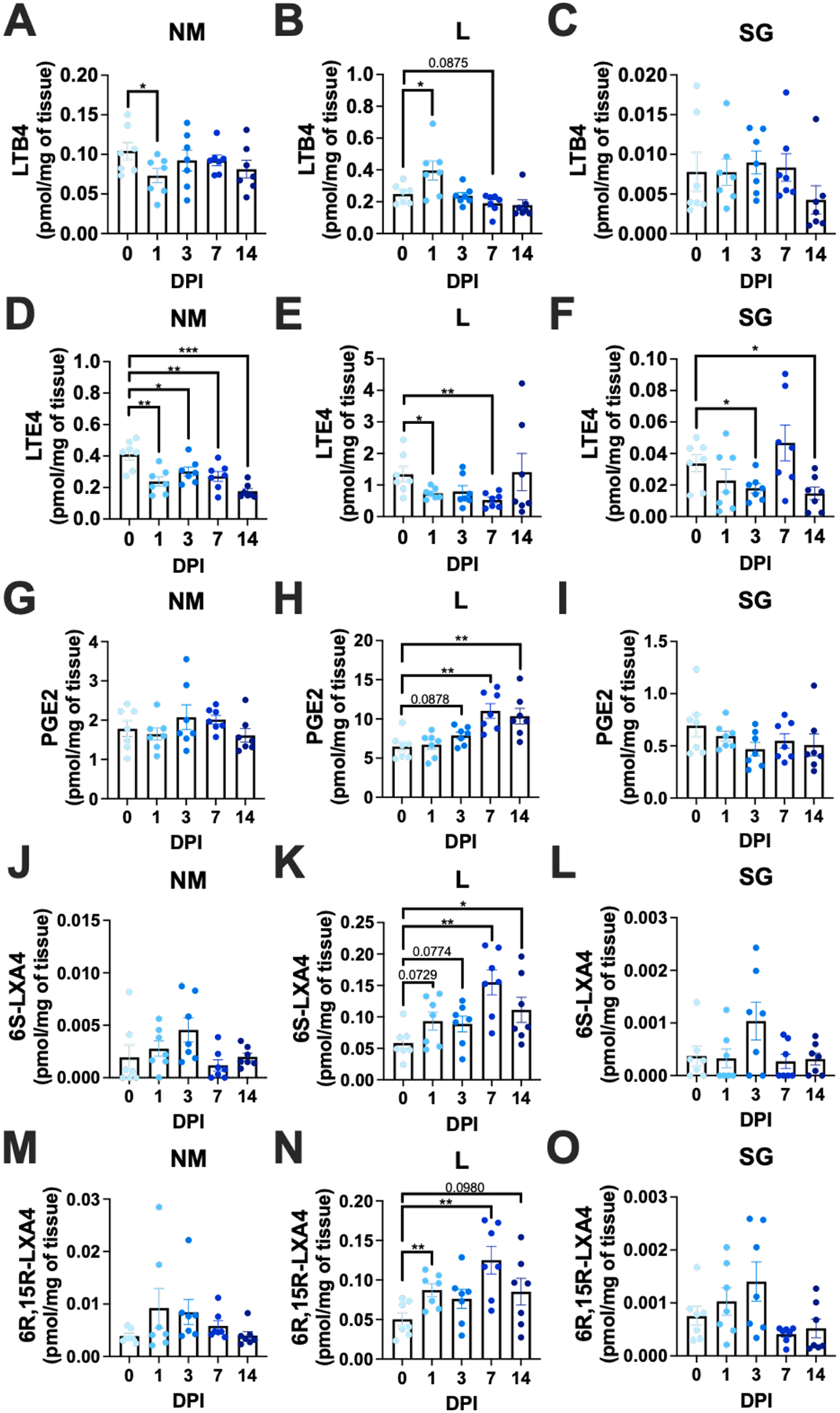
AA derived lipid mediators produced at steady state and throughout MCMV infection in the NM, lung and SG. Tandem mass spectrometry quantification of (A-C) LTB4, (D-F) LTE4, (G-I) PGE2, (J-L) 6S-LXA4, and (M–O) 6R, 15R-LXA4 at 0, 1, 3, 7 and 14 DPI. Shown are results from the NM, lungs, and SG of B6 i.n. infected mice. Data graphed as a scatter dot bar plot showing individual values of each mouse and mean with SEM. n = 7 mice per time point. (Brown-Forsythe and Welch One-way ANOVA) * = p ≤ 0.05, ** = p ≤ 0.01, *** = p ≤ 0.001.

Lipid mediator production varies in response to different inflammatory stimuli and in different tissues of the body^99–102^ and is almost entirely unknown in the context of CMV infection. We infected WT mice by the i.n. route and used tandem mass spectrometry to quantify a panel of 14 pro-inflammatory and pro-resolving lipid mediators in the NM, lungs, and SG over time (Fig. 2 & S2). The quantities of AA-derived leukotrienes, prostaglandin E2 (PGE2) and lipoxin A4 (LXA4) enantiomers are shown in (Fig. 2A–O) while the pro-resolving lipids derived from DHA and EPA are shown in (Fig. S2A–I). Most of the lipids were present at steady state in the NM and lungs of uninfected mice. In contrast, while the SG of uninfected mice also contained steady-state levels of the pro-inflammatory leukotrienes (Fig. 2C&F) and PGE2 (Fig. 2I), uninfected SG lacked most of the pro-resolving lipids, with the notable exceptions of LXA4 and resolvin E2 (RvE2, Fig. 2L&O and Fig. S2B). Interestingly, the quantities of each lipid differed markedly between tissues. For example, the AA-derived lipids LTB4, LTE4 and PGE2 were present in uninfected lungs at levels that were 2–3 times higher than in the NM, and 9–40 times higher than in the SG, and these differences were even greater for the LXA4 enantiomers (Fig. 2). In contrast, the DHA- and EPA-derived pro-resolving lipids tended to be present at higher levels in the NM compared to the lungs (with some exceptions) and were largely absent from the uninfected SG (Fig. S2). Thus, the balance of lipids appeared to be skewed towards a pro-resolving state in the NM relative to the lungs, while the SG expressed fewer lipids overall.

MCMV infection induced changes in lipid production that differed by organ and were less coordinated than expected. The lungs had significant increases in LTB4 (Fig. 2B), PGE2 (Fig. 2H) and LXA4 (Fig. 2 K&N), and significant reductions in resolvin E4 (RvE4) and maresin (Fig. S2C&F). However, none of this happened in the NM apart from LXA4 production, which trended upward but failed to reach significance (Fig. 2G, J, M & S2). Instead, infection induced a small but significant decrease in LTB4 production in the NM at 1 DPI (Fig. 2A), and a marked upregulation of resolvin D2 (RvD2), which did not occur in the lungs (Fig. S2E). Interestingly, one consistent outcome in both tissues was the significant decrease in production of the cysteinyl LTE4 after infection (Fig. 2D–E). Together, these data suggest that the NM became even more skewed towards a pro-resolving state after MCMV infection (increased RvD2 and decreased LTB4 and LTE4) while the lungs trended even further towards an inflammatory state (increased LTB4, PGE2, and reductions in RvE4 and maresin). In the SG, LTB4 and PGE2 levels remained unchanged throughout the time course while LTE4 levels declined (Fig. 2C, F & I). However, while some mice showed small increases in various pro-resolving mediators in the SG after infection, the effects were transient and did not reach significance, except for a loss of RvE2 over time (Fig. S2B). Thus, the SG did not trend towards either an inflammatory or consistently pro-resolving state during this acute infection window. Together, these data show that lipid mediator levels differ markedly at steady state between the NM, lungs, and SG. The NM tended to be more pro-resolving and the lungs more pro-inflammatory at baseline, and these profiles were exacerbated by MCMV infection, rather than coordinated across tissue environments by the introduction of a virus. We were most interested in the leukotrienes given prior work implicating these lipids in inflammation induced by CMV and their potential to promote viral control.^70–71,78–79^ Notably, the leukotrienes were present in all tissues at different baseline levels, and these levels were only subtly changed by infection.

### There is no direct role of 5-LO or leukotrienes on viral growth

Previous work suggested that LTB4 may directly impact HCMV replication,^78–79^ while the COX-2 enzyme, which processes AA into PGE2 and other inflammatory mediators, is known to be required for HCMV replication.^68–69,75,103–104^ Therefore, it was possible that 5-LO and/or LTB4 were directly inhibiting MCMV growth in the NM and lungs. To test this, ex vivo bone marrow derived macrophages (BMDMs) and primary fibroblasts were generated from WT and 5-LO^−/−^ mice and infected with MCMV for multistep growth curves. Virus grown in 5-LO^−/−^ cells showed no difference in growth compared to virus grown in WT cells (Fig. S3A&B). Moreover, daily treatment with a leukotriene mix containing LTB4, LTC4, LTD4, LTE4, LTF4, and N-acetyl LTE4 did not inhibit viral growth (Fig. S3C). Therefore, neither lack of 5-LO signaling nor leukotriene treatment altered viral growth directly in vitro.

### BLT1 deficiency leads to reduced IFN-γ production in the NM and lungs but unchanged IL-10 levels, altering the IFN-γ:IL-10 ratio

**Fig. 3.**
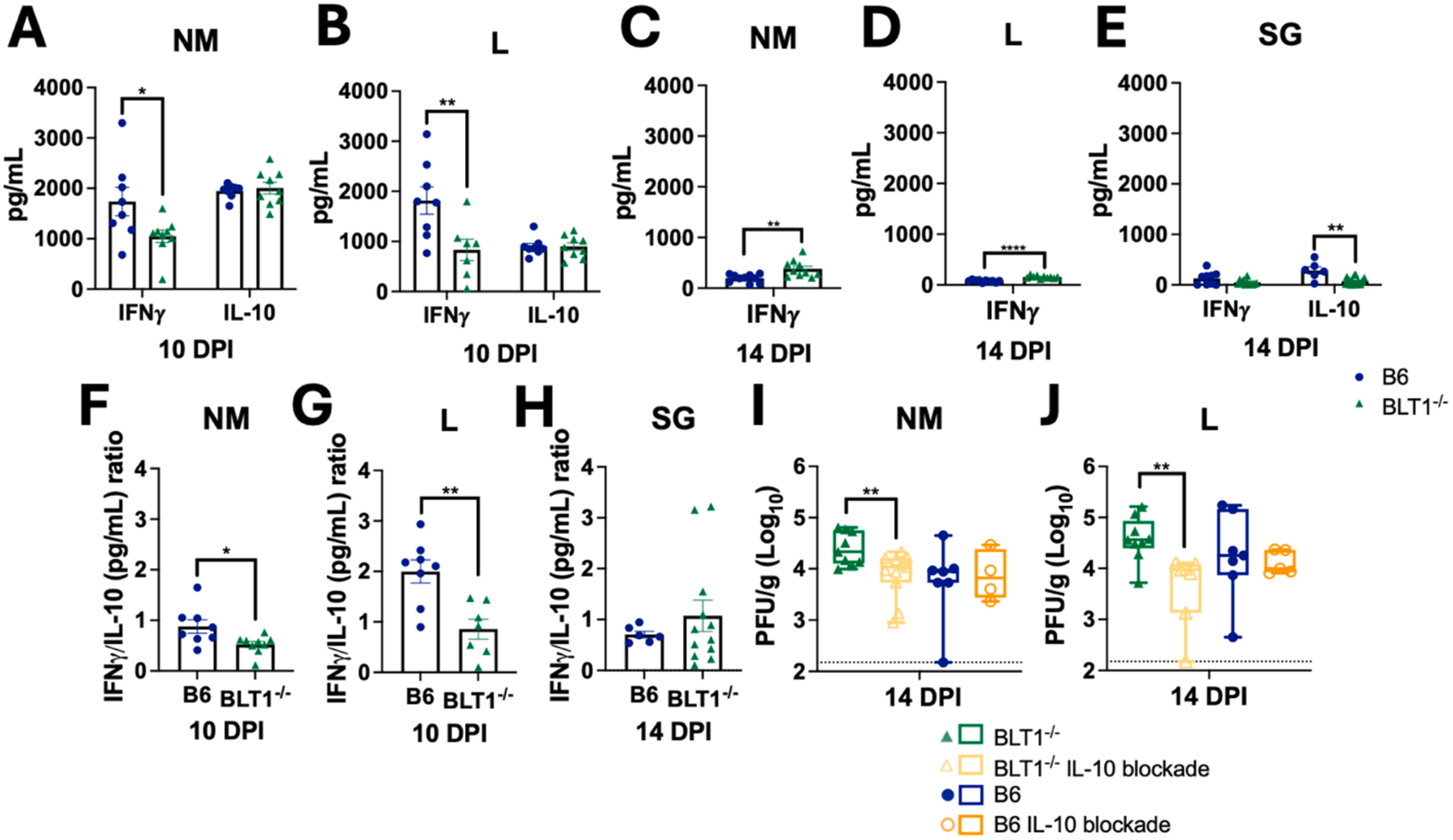
BLT1^−/−^ mice produce less IFNg in the NM and lungs, leading to a reduced IFNg:IL-10 ratio and IL-10-dependent increases in viral loads. ELISA quantification of IL-10 and IFN-γ in the NM and lungs at (A-B)10 DPI and (C-D) IFN-γ at 14 DPI of i.n. infected B6 and BLT1^−/−^ mice. (E) ELISA quantification of IL-10 and IFN-γ in the SG at 14 DPI of i.n. infected B6 and BLT1^−/−^ mice. Ratios of IFN-γ:IL-10 in the (F) NM, (G) lungs at 10 DPI and (H) SG at 14 DPI determined by dividing IL-10 production from IFN-γ production. (I-J) Viral loads as assessed by plaque assay in WT and BTL1^−/−^ mice with or without blockade of the IL-10 receptor. Individual values represent a single mouse ranging from 4 to 11 mice per group from at least two independent experiments. (Mann-Whitney test: A-E, Welch’s *t* test: F-H, Welch’s *t* test on log transformed data I-J) *p < 0.05; **p < 0.01, ***p < 0.001.

IFN-γ is essential for control of CMV.^30,35–36,105–114^ Extensive work in mice has shown that the rate of viral control in the SG is controlled by the balance of IFN-γ, which promotes viral control, and IL-10, which enables viral persistence.^27,35,115–118^ Specifically, blockade of IL-10 signaling, or promotion of IFN-γ, improves viral control,^27,115–116^ and increased levels of IL-10 drive increased viral loads.^116^ Strikingly, infected BLT1^−/−^ mice produced significantly less IFN-γ in the NM and lungs at 10 DPI compared to WT mice (Fig. 3A–B). This was not due to a delay in IFN-γ production in BLT1^−/−^ mice since IFN-γ levels declined in the NM and lungs of all mice by 14 DPI (Fig. 3C–D). Notably however, IFN-γ levels didn’t drop quite as much in the BLT1^−/−^ mice, resulting in slightly more IFN-γ remaining at 14 DPI in the NM and lungs. This could be due to the elevated viral loads in BLT1^−/−^ mice at this time (Fig. 1B). In contrast to IFN-γ, IL-10 was unchanged in the NM and lungs (Fig. 3A–B). In the SG, it has been demonstrated that a higher IFN-γ:IL-10 ratio promotes viral control.^115^ Importantly, the unchanged IL-10, coupled with the reduced IFN-γ resulted in a significant reduction in the IFN-γ:IL-10 ratio in the NM and lungs of BLT1^−/−^ mice (Fig. 3F&G). Thus, loss of BLT1 alters the IFN-γ:IL-10 ratio in key mucosal sites of viral entry. In the SG levels of IFN-γ and IL-10 were both low at these early times (MCMV arrives in the SG between days 7 and 14 after i.n. infection.^22^ SGs of WT mice produced an average of 127 pg/mL of IFN-γ and 286 pg/mL of IL-10, which were reduced by 2.3x and 3.8x respectively in BLT1^−/−^ mice (Fig. 3E). Reductions in both cytokines in led to a comparable IFN-γ:IL-10 ratio (Fig. 3H) in this tissue where viral loads were unaffected by BLT1-deficiency (Fig. 1B).

### Blockade of IL-10R restores viral control in the NM and lungs

Previous work showed that increased IFN-γ or blockade of the IL-10 receptor can promote control of MCMV in the SG of WT mice,^27,115–116^ while increased IL-10 drove more MCMV replication in the SG.^116^ In contrast, we have previously shown that blockade of the IL-10 receptor had no effect on MCMV titers in the NM or lungs of WT mice.^87^ However, the reduced IFN-γ production and altered IFN-γ:IL-10 ratio in BLT1^−/−^ mice led us to speculate that there could be an increased impact of IL-10 in these tissues. Indeed, blockade of the IL-10 receptor in BLT1^−/−^ mice restored viral titers in both the NM and lungs to levels that were comparable to those in WT mice (Fig. 3 I&J). Treatment of WT mice with IL-10 receptor blockade had no further effect (Fig. 3 I&J), consistent with our previous work.^87^ Together, these data show that LTB4 signaling through the BLT1 receptor is required for optimal IFN-γ production, which is required to overcome IL-10 in the NM and lungs to enhance viral control.

### BLT1 deficiency causes only subtle alterations in the cellularity of MCMV-infected tissues but impacts T cell differentiation

Next, we examined the immune cell infiltration in the NM, lungs, and SG of WT and BLT1^−/−^ mice using flow cytometry, and the gating strategies outlined in Fig. S4. There were significant changes in some of the granulocyte and dendritic cell populations during early infection, but these were not consistently reduced across all tissues or at similar timepoints (Fig. S5A). Furthermore, there were no differences between the size of the infiltrating T cell populations (Fig. S5B). Thus, loss of BLT1 only leads to subtle changes in the cellularity of the infected NM, lungs and SG.

**Fig. 4.**
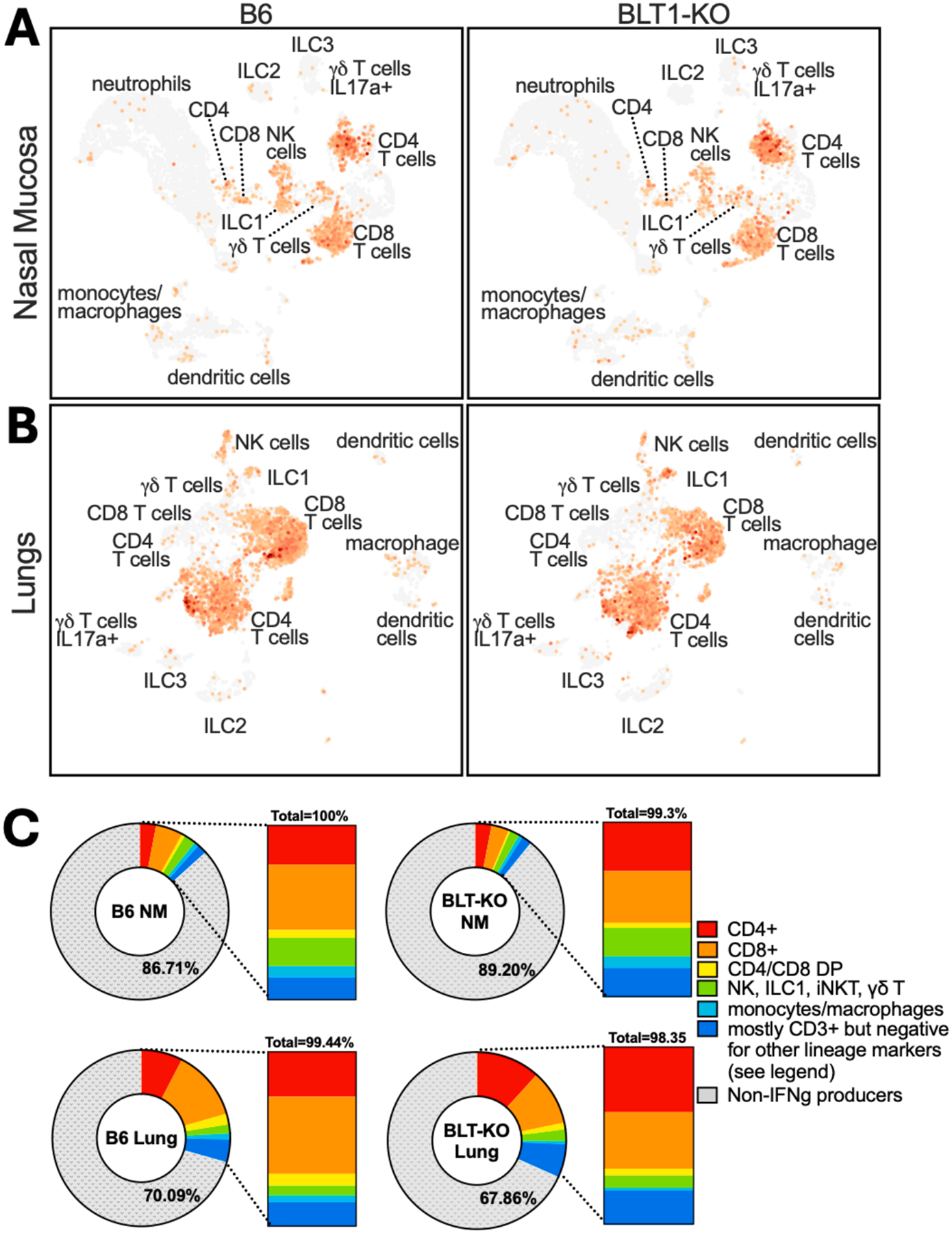
T cells are the primary IFN-γ-producing cells in the NM and lung. Single-cell RNA-seq was performed on sorted CD45^+^ cells from the NM and lung. Cell types were defined as described in the methods and assessed for expression of IFN-γ. Shown are the overall clusters and cell types of IFN-γ-expressing cells in the (A) NM and (B) lungs. (C) Quantification of the cell-types producing IFN-γ in both tissues is graphed to show the frequency of IFN-γ-producing cells (colored sections of the circles) among all cells in each tissue and within all IFN-γ producers (stacked bar graphs). Numbers in the circles indicate the percentage of cells that did not express IFN-γ. Numbers on top of the stacked bar graph indicate the sum of all subsets as a percentage of all IFN-γ producers. For simplicity, IFN-γ+ innate lymphocytes expressing either Zbtb16, Ncr1 or Trdc were pooled together (green). An undefined population of cells was also identified that produced IFN-γ (darker blue). These cells were Zbtb16^−^, Ncr1^−^, Trdc^−^, CD14^−^, Adgre1^−^, Fcgr1^−^, Fcgr3^−^, CD4^−^, CD8a^−^, CD8b1^−^. Approximately half of these undefined cells expressed CD3.

To get more detail about the cells responding to infection, we performed CITE-seq focused on immune cells that reached the NM and lungs. An example of the data quality analyses is shown in Fig. S6. Clusters of cell types were defined based on gene expression profiles as described in the materials and methods and major cell types were confirmed by antibody-derived tags confirming protein expression (Fig. S7&S8). Interestingly, the cell types that expressed IFN-γ were broadly similar between WT and BLT1^−/−^ mice (Fig. 4A–B). Moreover, in both cases, the majority of IFN-γ-producers were CD4^+^ and CD8^+^ T cells, with NK cells, γ/δ T cells and ILC1s also contributing a percentage of the IFN-γ at 14 DPI. However, quantifying the proportion of the various cell types producing IFN-γ revealed a reduced contribution from CD8^+^ T cells in BLT1^−/−^ mice, which was subtle in the NM and obvious in the lung (Fig. 4C).

**Fig. 5.**
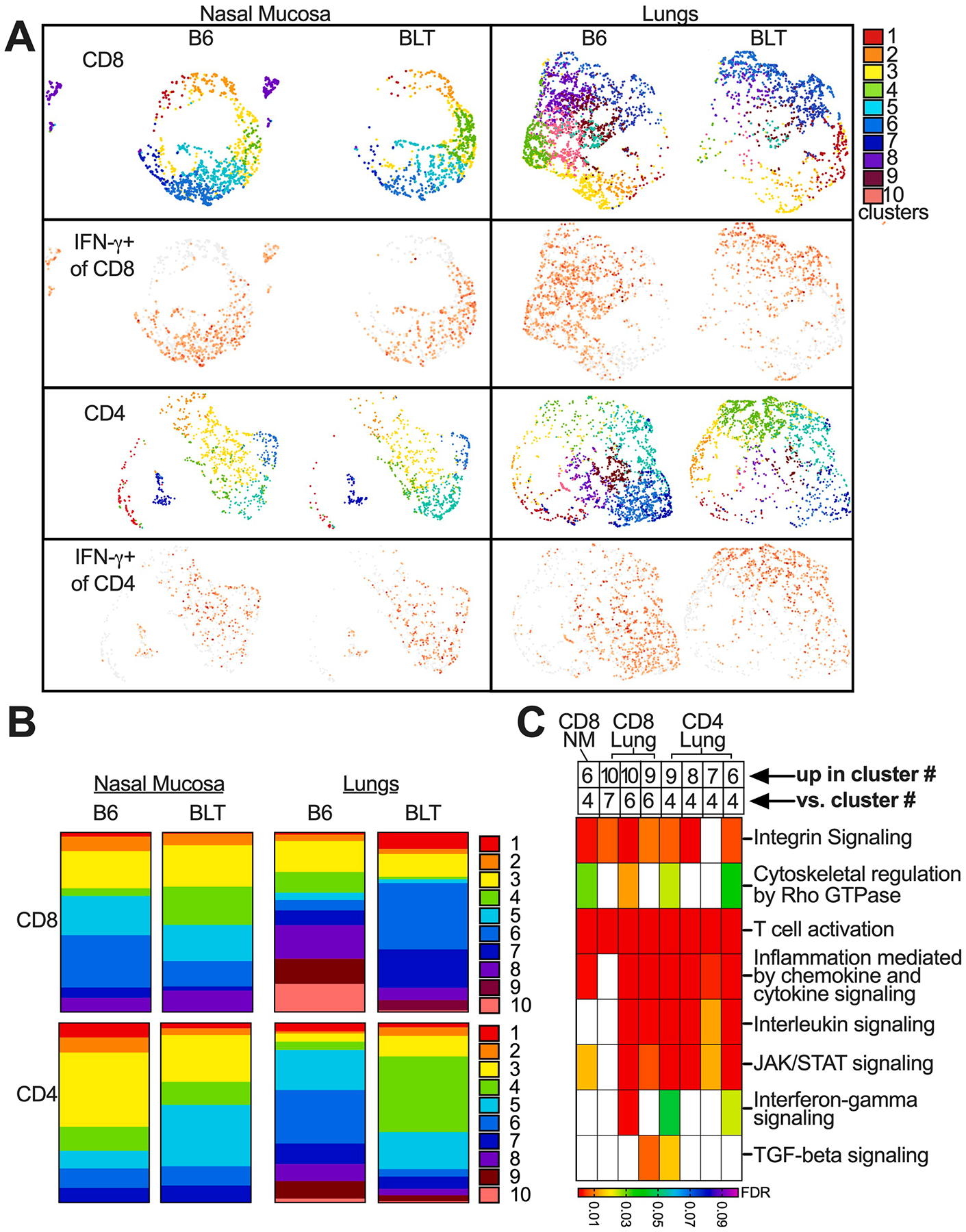
BLT^−/−^ T cells are enriched in different clusters from WT T cells, especially in the lungs. (A) CD8^+^ or CD4^+^ T cells as defined in the methods were re-clustered and expression of IFN-γ was defined across all clusters. (B) Stacked bar graphs show the frequency of cells within each cluster. (C) Clusters that were relatively enriched in B6 T cells were compared to clusters enriched in BLT^−/−^ T cells. Genes that were significantly upregulated (adjusted p-value < 0.2) in the indicated B6 T cell enriched clusters vs. BLT^−/−^ T cell enriched clusters were assessed for overrepresentation within Panther pathways. The heat map shows the False Discovery Rate (FDR) for the indicated pathways identified as overrepresented among upregulated genes in B6 T cell enriched clusters. Unfilled boxes had no significant enrichment of the indicated pathways.

Next, we isolated CD4^+^ and CD8^+^ T cells (Fig. 5A). Differential gene expression analyses revealed very few genes that differed between WT and BLT1^−/−^ CD4^+^ or CD8^+^ T cells in the NM (Table S1). In the lungs, wild-type CD8^+^ T cells expressed significantly increased levels of multiple histones and *Cdk8*, possibly indicating increased proliferation. Moreover, there were significant increases in multiple genes associated with IFN signaling or regulation (e.g. *Stat1*, *Nlrc5, Trim30a, Irak2, Ifi203, Card11*, Table S1). Wild-type CD4^+^ T cells in the lung did not express higher levels of the histones, but did show significantly increased upregulation *Cdk8, Stat1, Nlrc5,* and *Ifi203* along with increases in *Itga4*, *CCR5* and *Tox* (Table S1). Fewer genes were upregulated in BLT^−/−^ T cells compared to WT T cells (Table S1). Interestingly however, these analyses revealed clusters that were substantially under-represented in BLT1^−/−^ mice compared to WT mice (Fig. 5A–B). Again, differences were subtle in the NM, but obvious in the lungs. Notably, expression of IFN-γ mirrored these shifts, with the majority of WT IFN-γ-expressing T cells present in clusters that were under-represented in BLT1^−/−^ mice (Fig. 5B). In contrast, there were no differences in IL-10 expression in these cells, mirroring our ELISA findings (Fig. S9). These data indicate that IFN-γ-producing T cells, and indeed T cells in general, in BLT1^−/−^ mice differed from their WT counterparts in their gene expression profiles.

Assessment of differentially expressed genes between WT and BLT1^−/−^ T cells within individual clusters revealed very few genes that were significantly differentially expressed (i.e. most T cells in a cluster shared a similar transcriptional signature, as expected). Thus, to better define the differences between T cells in WT and BLT1^−/−^ mice, we performed over-representation analyses between clusters. To this end, clusters that were enriched with WT T cells (e.g. lung CD8^+^ T cell clusters 8, 9 and 10 and lung CD4^+^ T cell clusters 6, 7, 8, 9) were compared to clusters that were enriched in BLT1^−/−^ T cells (e.g. lung CD8^+^ clusters 6 and 7, and lung CD4^+^ cluster 4) (Fig. 5B). Genes identified as upregulated (adjusted p-value < 0.2) in clusters enriched in WT T cells were assessed for pathway overrepresentation by the PANTHER Pathway database.^94^ These analyses revealed that clusters enriched in WT T cells differed by integrin signaling, cytoskeleton regulation by Rho GTPases, T cell activation, and cytokine/chemokine signaling, compared to clusters enriched in BLT1^−/−^ T cells (Fig. 5C, Table S2). This was true among CD8^+^ and CD4^+^ T cells in the lung and among CD8^+^ T cells in the NM. Expanding these analyses using the Reactome pathway database^95^ revealed the same themes: marked enrichment of Rho, Rac and Cdc42 pathways in all clusters enriched in WT T cells compared to clusters enriched in BLT1^−/−^ T cells, as well as many pathways involved in T cell activation and cytokine or chemokine signaling (Fig. S10, Table S3). We further isolated and re-clustered IFN-γ-expressing cells to determine whether these differences were dependent on T cell activation state. Once again however, comparisons between clusters revealed the signature of Rho, Rac and Cdc42 pathways in clusters that were enriched in WT T cells (Table S3). Thus, lack of BLT1 signaling significantly impacted T cell expression of Rho, Rac and Cdc42 pathways, as well as T cell activation and cytokine signaling.

### BLT1 is not necessary for T cell chemotaxis to the NM, lung or SG

**Fig. 6.**
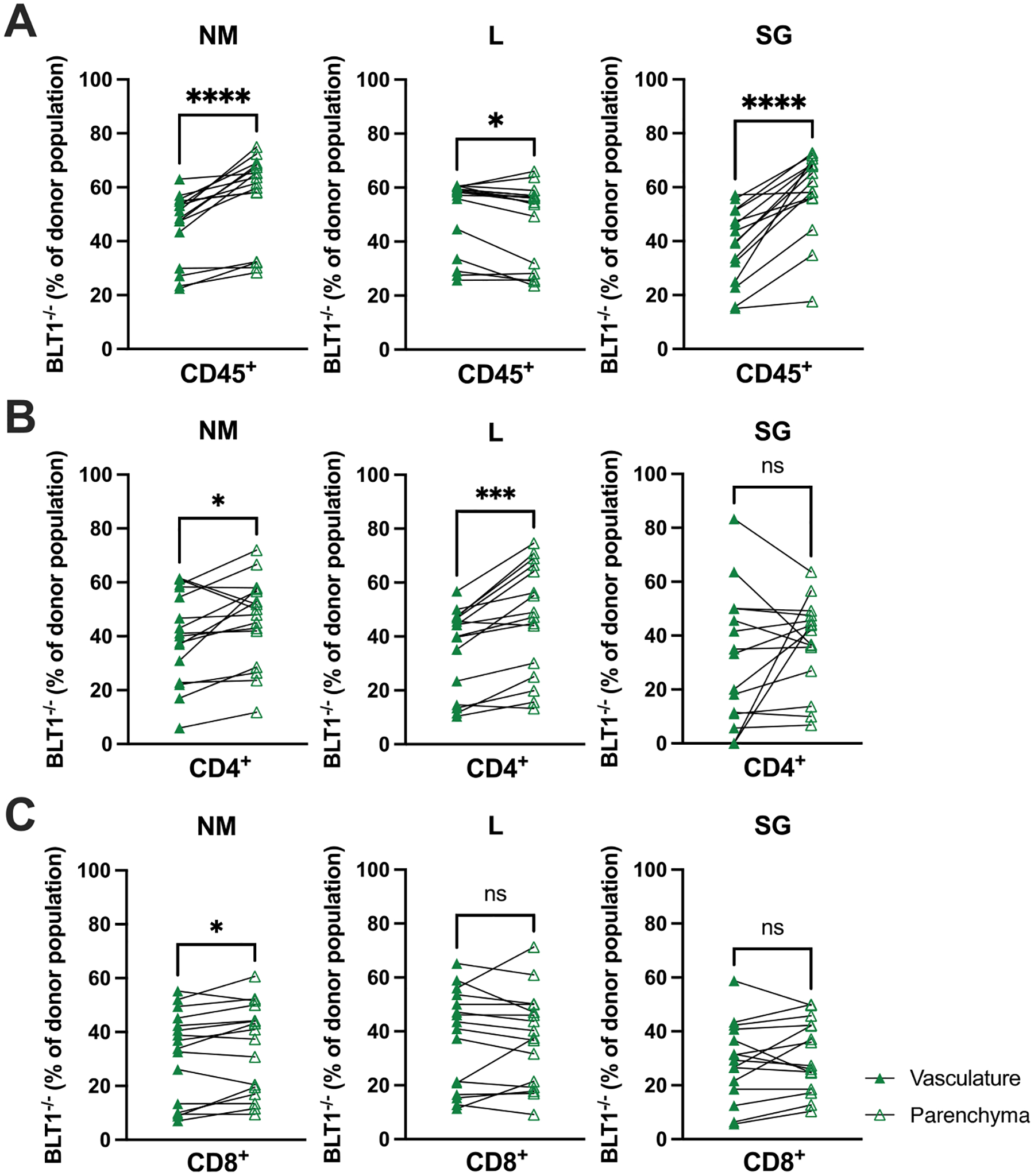
BLT1 is not required for recruitment of T cells to the NM and lungs. Mixed bone marrow chimeras were infected with MCMV. Shown are the frequencies of BLT1^−/−^ (CD45.2) cells as a proportion of all donor (A) CD45^+^, (B) CD4^+^ and (C) CD8^+^ cells in the vasculature or parenchyma of the NM, lungs and SG. Data are from 14 DPI of 15–16 mice per group from three independent experiments. (Wilcoxon test) *p < 0.05; **p < 0.01, ***p < 0.001.

The Rho, Rac and Cdc42 pathways modulate the cytoskeleton, cell motility and integrin signaling.^119^ Leukotriene B4 is a well-known chemotactic factor for immune cells, which has been best described for neutrophils,^120–122^ but also described for T cells.^123–126^ Thus, these data suggest that loss of BLT1 may have altered the T cell migratory potential and possibly activation by antigen. To explore the role of BLT1 in guiding T cell responses to MCMV infection, we generated mixed bone marrow chimeras by transferring mixtures of WT (CD45.1) and BLT1^−/−^ (CD45.2) bone marrow into irradiated TCR-β^−/−^ recipient mice (Fig. 6). In this way, all T cells will be derived from the donor mice, and we will be able to directly compare WT and BLT1^−/−^ T cells in the same environment. Based on pilot experiments, we transferred 2.5-times more BLT^−/−^ bone marrow to achieve equal amounts of WT and BLT^−/−^ T cells in recipient mice (Fig. S11). Then, after reconstitution, recipients were infected, and intravascular (i.v.) staining was used at 14 DPI to determine whether WT (CD45.1^+^) had a selective advantage in migrating into the parenchyma of each tissue relative to BLT1^−/−^ (CD45.2^+^) T cells. If BLT1 is a key chemotactic agent responsible for recruiting T cells to the NM, lungs or SG, we would expect the BLT1^−/−^ donor cells to have a selective disadvantage in migrating out of the blood to reach the tissue parenchyma. Strikingly, however, the migration of BLT1^−/−^ T cells into tissues was not impaired. Using the frequency of BLT1^−/−^ cells in the vasculature as a baseline benchmark for each mouse showed that BLT1^−/−^ cells were either equally able or better at reaching the parenchyma of the NM, lungs and the SG of infected mice (Fig. 6). These data suggest that BLT1-directed chemotaxis does not contribute to T cell migration to, or retention within the NM, lungs or SG after MCMV infection.

### BLT1^−/−^ CD8^+^ T cells can produce similar levels of IFN-γ compared to WT CD8^+^ T cells

**Fig. 7.**
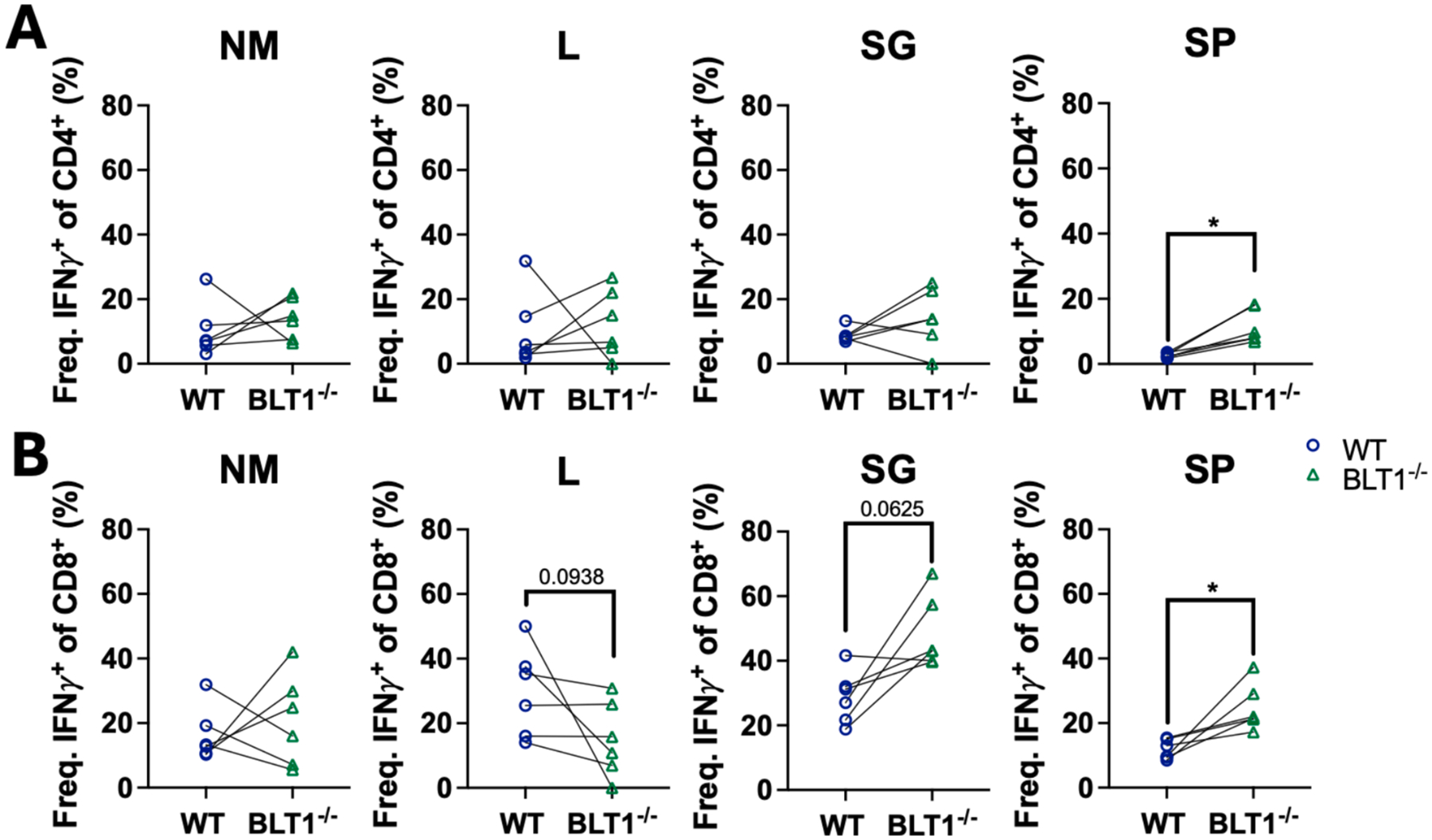
BLT1^−/−^ and WT T cells from mixed bone marrow chimeras produce similar amounts of IFN-γ. Lymphocytes from NM, lungs, SG, and spleen (SP) of mixed bone marrow chimera mice were isolated 14 DPI and stimulated with PMA/Ionomycin. IFN-γ production was analyzed by intracellular flow cytometry for (A) CD4^+^ and (B) CD8^+^ T cells from WT (45.1) and BLT1^−/−^ (45.2) donors. Data are from 6 mice per group from three independent experiments. (Wilcoxon *t* test) *p < 0.05; **p < 0.01, ***p < 0.001.

Next, we tested whether BLT1^−/−^ T cells that reached the parenchyma, and their WT counterparts, were equally functional. First, T cells were sorted from the parenchyma (i.v. staining-negative) of the mixed bone marrow chimeras and assessed for IFN-γ and IL-10 transcription by RT-qPCR. No significant differences in the expression of either cytokine were detected between WT and BLT1^−/−^ CD4^+^ or CD8^+^ populations (Fig. S12). Additionally, we stimulated T cells from mixed bone marrow chimeras with PMA and ionomycin and assessed IFN-γ production by intracellular cytokine staining. Again, parenchyma-localized BLT1^−/−^ T cells (both CD4^+^ and CD8^+^) produced similar or even greater amounts of IFN-γ in response to stimulation (Fig. 7). Thus, BLT1 deficiency did not impair the ability of T cells to produce IFN-γ on a per cell basis.

### BLT1^−/−^ T cells are poor at competing for access to antigen

**Fig. 8.**
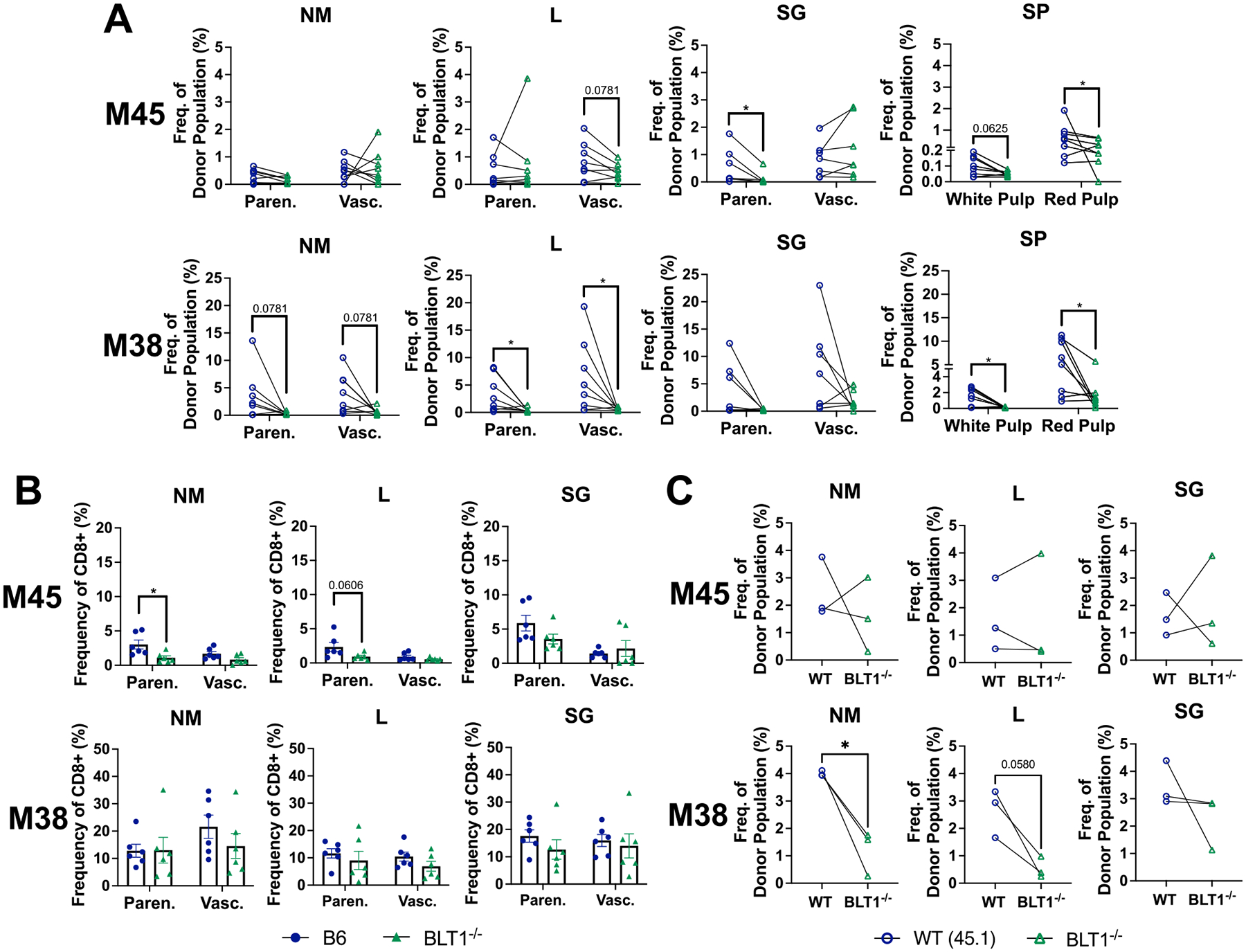
BLT1^−/−^ T cells are capable of producing antigen-specific CD8^+^ T cell responses but are poor at competing for access to antigen. (A) Lymphocytes from mixed bone marrow chimera mice were isolated from the indicated tissues at 14 DPI and analyzed by flow cytometry for M45- and M38-specific CD8^+^ T cells. Tetramer^+^ T cell populations are shown as percentages of the donor population in the parenchyma or vasculature of the indicated organs. Lines connect the WT and BLT1^−/−^ T cells from the same chimeric mice. (B) Lymphocytes from B6 and BLT1^−/−^ mice were isolated at 14 DPI and analyzed by flow cytometry for M45- and M38-specific CD8^+^ T cells. The tetramer^+^ T cell populations are shown as percentages of CD8^+^ T cells in the indicated strain. (C) Lymphocytes from NM, lungs, and SG of mixed bone marrow chimera mice were isolated 14 DPI and stimulated with M45 and M38 peptides. IFN-γ production was analyzed in all recovered T cells (not fractionated by i.v. staining) by intracellular cytokine staining for WT (45.1) and BLT1^−/−^ (45.2) donors. Data are from 3 to 8 mice per group from at least two independent experiments. (Wilcoxon test: A, Mann-Whitney test: B, Paired *t* test: C) *p < 0.05; **p < 0.01, ***p < 0.001.

Prior work from our lab and others has shown that competition for infected targets is a key driver of MCMV-specific T cell expansion after the first week of infection.^37,44–45,127^ The kinetics of this have been well-defined. M45-specific T cells are the most abundant population primed by infection, but then contract after the first week of infection due to an absence of viral antigen on infected cells.^39,128–129^ In contrast, M38-specific T cells undergo persistent antigen stimulation and cell division and become a dominant population by the second week of infection.^39–40,43^ Importantly, persistent stimulation and expansion of M38-specific T cells is very sensitive to competition: failure to access infected targets severely impairs responses by M38-specific T cells.^44–45,127^ Thus, T cells that successfully compete for antigen expand, while unsuccessful T cells do not, unless the competition is removed.^37,44–45^ If BLT1^−/−^ T cells struggle to compete for access to antigen compared to WT T cells, we would expect a lack of BLT1^−/−^ M38-specific T cells when there is competition between WT and BLT1^−/−^ T cells. Strikingly, this was the case: in chimeric mice, where WT and BLT1^−/−^ T cells are in direct competition, M38-specific responses were significantly reduced among the BLT1^−/−^ T cell population in both the parenchyma and vasculature fractions (Fig. 8A,C), as assessed by both tetramer staining and cytokine production after peptide stimulation. In contrast, T cells specific for the viral M45 antigen were generally similar between WT and BLT^−/−^ T cell populations. If this lack of M38-specific T cells from the BLT1^−/−^ donor is due to competition for antigen, we would expect an improved expansion of M38-specific T cells in intact BLT1^−/−^ mice, where they do not face any competition from WT T cells. Indeed, M38-specific T cell frequencies were nearly comparable between infected WT and BLT1^−/−^ mice at 14 DPI (Fig. 8B) as well as earlier at 10 DPI (Fig. S13). These data suggest that BLT1^−/−^ T cells are primed normally but are poor at competing for access to infected cells for persistent stimulation and are thus outcompeted by their WT counterparts. In line with this, we found that expression of the proliferation marker Ki-67 was substantially reduced among BLT1^−/−^ CD8^+^ T cells in the lungs of BLT1^−/−^ mice in our scRNA-seq data set (Fig. S14). These data support the notion that BLT1^−/−^ T cells are inefficient at encountering infected target cells, leading to reduced T cell proliferation and activation. Together, these data suggest that leukotriene B4 promotes control of MCMV by optimizing T cell encounters with infected targets, maintaining the balance between IFN-γ and IL-10.

## Discussion

Our data reveal a novel mechanism underpinning the control of MCMV in mucosal sites of entry and persistence. Specifically, we suggest that the eicosanoid LTB4, via its receptor BLT1, drives control of MCMV by optimizing T cell encounters with infected targets, thus promoting sufficient levels of IFN-γ to overcome local IL-10 in the NM and lungs. These data highlight the overlooked role of bioactive lipid mediators in optimizing anti-viral responses. Importantly, we also provide the first map of lipid mediator production in critical sites of CMV entry and shedding, showing a remarkable degree of diversity in different tissues between lipid mediator production at baseline and after infection by MCMV.

Lipid mediators, like cytokines, are potent signaling molecules and crucial components of the inflammatory response.^52–53,48–50^ They are produced by resident cells in either injured or infected tissues, and released from cells to act in both autocrine and paracrine fashions by binding to their appropriate g-protein-coupled receptors on various cell types.^52–53,99,48–50^ Their effects are complex and impact many pathways, including inflammation, vasodilation, cell trafficking and function, and mucus production.^52–53,48–50^ Interestingly, the role of a single lipid mediator can differ from one infection to another, demonstrating the importance of mapping out the roles of lipid mediators in each individual infection.^49,130^ Even so, the functional impact of lipid mediators in pathogenic infections, particularly viral infections, remains largely unexplored. For example, lipid mediators were detected in the bronchiolar lavage and nasal lavage fluids from patients with COVID-19,^131–132^ bronchiolar lavage fluid and nasal washes of mice and humans infected with influenza,^133^ and nasal lavage fluids from infants with respiratory syncytial virus-induced bronchiolitis.^134^ However, it remains largely unknown how these lipids influence viral immunity and disease in each case. Inhibition of LTB4 synthesis in patients with COVID-19 was unable to reduce the duration of symptoms.^135^ Moreover, previous work suggested that LTB4 could enhance Type I IFN levels and pulmonary neutrophil responses in influenza-infected mice, correlating with improved outcomes of infection.^136–137^ However, while mice lacking BLT1 have increased mortality after influenza infection, this was traced to increased immune pathology rather than altered viral control.^138^

In the case of CMV, very little is known about the lipid mediators produced in vivo after infection. HCMV replication in infected cells in culture is known to require COX-2 expression for the production of PGE2,^68–69,75,103–104^ and PGE2 directly upregulates the HCMV major immediate early promoter in macrophages.^104^ Additionally, HCMV infection of various cell lines upregulates 5-LO and COX-2 expression^68–71^ and can increase the production of LTB4.^71^ Interestingly, we found that leukotrienes and PGE2 were present at steady state in the NM, lungs, and SG, with quantities in the lung being markedly higher than the NM or SG (Fig. 2). Unexpectedly, only the lung increased production of LTB4 and PGE2 after infection (Fig. 2), despite the in vitro evidence that HCMV promotes both PGE2 and LTB4.^75,103–104,68–71^ These data may suggest that the infected cell type and the environment strongly influence the in vivo production of lipids. Moreover, the data imply that the lung environment is more poised than the NM or SG to produce inflammatory lipids, at least after MCMV infection. Importantly, the production of pro-inflammatory eicosanoids is thought to be temporally regulated, with pro-inflammatory lipids produced early, followed by lipid mediator class switching to specialized pro-resolving mediators later in the response.^139^ These pro-resolving mediators actively resolve inflammation by, for example, stopping neutrophil recruitment, reducing inflammatory cytokines and T cell activation, and enhancing macrophage clearance of debris.^52–53^ Interestingly, most of the pro-resolving mediators in our panel were also present at steady state in the NM and lungs, with quantities in the NM being higher than in the lungs in general (Fig. S2). Thus, the NM appeared to be poised in a pro-resolving state while the lungs were more pro-inflammatory. Surprisingly, MCMV largely enhanced these differences, and evidence of lipid class switching after infection in these tissues was fairly limited. Of the pro-resolving mediators, only LXA4 in the lungs (Fig. 2 K&N), and RvD2 in the NM (Fig. S2E), were clearly elevated over time after infection. In contrast, most pro-resolving mediators were absent from the SG at steady state, and while a few appeared to be induced by infection, the mouse-to-mouse variability was quite high (Fig. 2, Fig. S2). It is possible that these relatively stable lipid-mediator profiles in each tissue reflect their microbiomes, or the specific environmental exposures experienced by each tissue. It is also possible that a relatively non-inflammatory virus like MCMV fails to markedly perturb this balance and/or that ongoing MCMV replication impairs the switch to a more generalized pro-resolving state in each tissue. Future work will be needed to address these issues.

Mucosal surfaces serve as the site of entry for many respiratory and intestinal pathogens, but the NM is relatively understudied despite being the first tissue encountered by many viruses.^140^ It is now appreciated that the NM, accessible from the mouth through the nasopharynx, serves as a natural site of entry for MCMV after oral/nasal exposure and a site of HCMV infection.^13–19^ However, mechanisms regulating immune function in this barrier tissue remain poorly understood. In contrast, an abundance of research has investigated the immunological response to MCMV in the SG, a major site of viral shedding. It is now well-known that the balance between IFN-γ and IL-10, both produced primarily by T cells in the SG, dictates control of MCMV.^27,106,33–35,115–118,141–145^ Specifically, increasing IFN-γ via OX40-agonism, or decreasing IL-10 signaling by IL-10 receptor blockade both improved viral control,^87,115^ while increased IL-10 resulted in increased viral replication.^116^ Interestingly, deficiency of 5-LO also led to elevated viral loads in the SG after intravenous infection (which enables direct infection of the salivary gland from the blood) and LTB4 treatment of these mice suppressed MCMV reactivation in an ex vivo assay.^79^ Although no mechanism was previously described for these results, these data suggest that lipid mediators also play a role in regulating viral control.

We previously showed that MCMV could replicate persistently in the NM, and that viral titers were regulated by T cells.^22,87^ However, in contrast to the SG, we found that IL-10 receptor blockade had no effect on viral titers in the NM or lungs of WT mice.^87^ Our new data show that 5-LO deficiency also impairs viral control in the NM and lungs (Fig. 1), and we traced this phenotype to the need for LTB4/BLT1 signaling to promote optimal IFN-γ production in the NM and lungs (Fig. 3). Perhaps most significantly, we found that control of MCMV in the NM and lungs of BLT1^−/−^ mice could be corrected by IL-10 blockade (Fig. 3I–J), despite no impact of BLT1 on IL-10 levels and no impact of IL-10 blockade in WT mice that make normal levels of IFN-γ (Fig. 3). These data suggest that viral control requires sufficient levels of IFN-γ to overcome local IL-10. In WT mice these levels are sufficient, but the reduction of IFN-γ in BLT1^−/−^ mice allowed IL-10 to modulate protective immune responses.

The role of these pathways in viral control in the SG appears to be different. While our data recapitulated the elevated viral loads in the SG of the 5-LO^−/−^ mice at 14 DPI, loss of BLT1 did replicate this phenotype (Fig. 1B), nor alter the IFN-γ/IL-10 ratio in the SG. The 5-LO enzyme is responsible for producing all leukotrienes and many other bioactive lipid mediators, and the precursors for these lipids will be shunted into other pathways when 5-LO is missing.^80–82^ Thus, the 5-LO^−/−^ mice have a complex phenotype and future work will be needed to dissect the role of 5-LO in promoting viral control in the SG.

Little is known about the effects of BLT1 signaling on T cell function. It was shown over 35 years ago that LTB4 could enhance IFN-γ secretion from human T cells in culture.^83^ Intriguingly, BLT-deficiency did not alter the ability of T cells to produce IFN-γ in vivo or ex vivo in our model (Fig. S12, Fig. 7). However, single-cell RNA sequencing results showed BLT1^−/−^ IFN-γ-producing T cells and T cells in general, differed from their WT counterparts by integrin signaling, cytoskeleton regulation by Rho GTPases, T cell activation, and cytokine/chemokine signaling (Fig. 5). Thus, these data suggest that BLT1^−/−^ T cells are functional, but that they may have altered migratory potential resulting in reduced activation by antigen. Cellular infiltration was critical to test since LTB4 is well-known to play a role in chemotaxis, particularly for neutrophil and monocyte populations,^120–122^ but also for effector T cells in some circumstances.^123–126^ Using mixed bone marrow chimeras, it was evident that BLT1^−/−^ T cells were recruited from the vasculature to the parenchyma normally (Fig. 6). However, our data strongly suggest that BLT1^−/−^ T cells were poor at competing for access to infected cells to receive antigen stimulation throughout the body. Indeed, prior work from the Oxenius lab showed that M38-specific T cells undergo persistent antigen stimulation and cell division, and we and others previously found that M38-specific T cells only expand if they can successfully compete for access to infected cells.^39–40,43–44,127^ Importantly, unsuccessful T cell clones and responses do not proliferate and expand unless the competition is removed.^37,44–45^ The mechanism for this was suggested by elegant work from the Reddehase lab, who showed previously that T cells recognizing viral antigen on infected cells silenced further viral transcription,^37^ thereby preventing any additional antigen production for other T cells to recognize. Thus, the fact that M38-specific T cells expanded normally in BLT1^−/−^ mice, but not in a mixed bone marrow chimera setting when in competition with WT T cells (Fig. 8), suggests that they are inefficient at accessing antigen. Consistent with this, BLT1^−/−^ T cells in the lung were undergoing substantially less proliferation compared to WT T cells based on expression of the proliferation marker Ki-67 (Fig. S14). Ultimately, MCMV was controlled in the 5-LO^−/−^ and BLT1^−/−^ mice (Fig. S1). Therefore, even these sub-optimal T cell responses were sufficient to eventually control MCMV, and the effect was only apparent at acute timepoints. Altogether, these results indicate that LTB4-BLT1 signaling optimizes T cell migratory potential and ability to encounter antigens, resulting in less migration to the lungs, less activation and proliferation, less IFN-γ production, and poor viral control.

We did not scrutinize the cell type(s) that are responsible for producing LTB4 after MCMV infection. Myeloid cells and mast cells are known to be key producers of LTB4 in other settings,^122,146–148^ but further studies are needed to elucidate this mechanism. However, our data imply that virus-infected cells are key sources of LTB4, which guides MCMV-specific T cells to their targets.

Our data do not rule out a role for BLT signaling in other cell types during MCMV infection. We focused on T cells due to the timing of the elevated viral loads and the prominent loss of IFN-γ. However, there were some subtle, but potentially interesting differences between BLT1^−/−^ mice and B6 mice regarding innate cellular populations of the NM, lungs, and SG. There were transient reductions in mast cells and basophils in the NM and lungs of BLT1^−/−^ mice and mast cells were nearly absent from the SG of BLT1^−/−^ mice (Fig. S5A). Mast cells are also known to contribute to MCMV immunity in the lung.^149^ However, the numbers of mast cells were restored in the NM and lungs by 14 DPI (Fig. S5A), so it is unclear whether this may have impacted viral control. Most interestingly, there was also a reduction in NK cell numbers in the SG of BLT1^−/−^ mice, and a trend towards reduced NK cell numbers in the lung (Fig. S5A). NK cells play critical roles in the early control of MCMV,^150–151^ and thus, any impact on NK cells is likely to have a significant impact on viral loads. Future work will be needed to determine whether BLT1-deficient NK cells are functionally impaired during MCMV infection.

It is also important to note that RvE1 has been shown to interact directly with the LTB4 receptor in human polymorphonuclear cells. In this capacity, RvE1 is suggested to play a regulatory role to control migration and cytokine production by locally dampening LTB4/BLT1 signals.^152^ We detected RvE1 levels in the NM and lungs before and after MCMV infection (Fig. S2A). Thus, further work will be needed to test the intriguing possibility that RvE1 might counteract LTB4 signaling on T cells in certain conditions, regulating viral control.

Collectively, these data raise the possibility that clinical agents that interfere with 5-LO or LTB4 signaling might reduce T cell activation and IFN-γ production, and thus pre-dispose patients to elevated CMV replication. Our study has important implications for patients on allergy medications that modulate the leukotriene pathway such as zileuton, which is an antagonist of 5-LO, montelukast, which modulates cysteinyl leukotrienes, and various LTB4 and BLT1 inhibitors, which are currently being assessed in clinical trials.^153^ Blockade of leukotrienes in these situations could play a role in making patients more susceptible to CMV infection or reactivation. However, LTB4 production may also promote disease in some settings. The ability of HCMV to activate 5-LO and LTB4 has been proposed to contribute to vascular inflammation and the development of atherosclerosis^71,154–155^. Moreover, the activation of COX-2 and 5-LO by HCMV has been proposed to act as a driver of cancer by mediating inflammation and angiogenesis.^76–77,72–74^ In addition, it will be important to determine how the balance between pro-inflammatory and pro-resolving mediators impacts viral loads, persistence, and pathogenesis by selectively targeting pro-resolving signaling pathways. Overall, our data indicate that BLT1 signaling is a critical component of an anti-viral inflammatory balance in the NM and lungs during CMV infection by optimizing T cell encounters with infected targets, thereby promoting sufficient IFN-γ production, to overcome local IL-10. More broadly, we show a remarkable degree of diversity in both the production of lipid mediators and the role of LTB4 in the NM, lungs and SG during infection with one single virus, emphasizing the significance of investigating lipid mediators and inflammatory responses to diverse viral infections and various relevant tissues.

## Supplementary Material

1

2

3

4

## Appendix A. Supplementary data

Supplementary data to this article can be found online at https://doi.org/10.1016/j.mucimm.2025.08.002.
