# Supplementary material for: Leukotriene B4 regulates T cell recognition and control of MCMV in mucosal tissues
Source: Mucosal Immunol. Author manuscript; Available in PMC 2026 Jan 19. (PMC12814996; doi:10.1016/j.mucimm.2025.08.002)
Supplement: 1 [file NIHMS2129402-supplement-1.pdf]

Supplementary Material

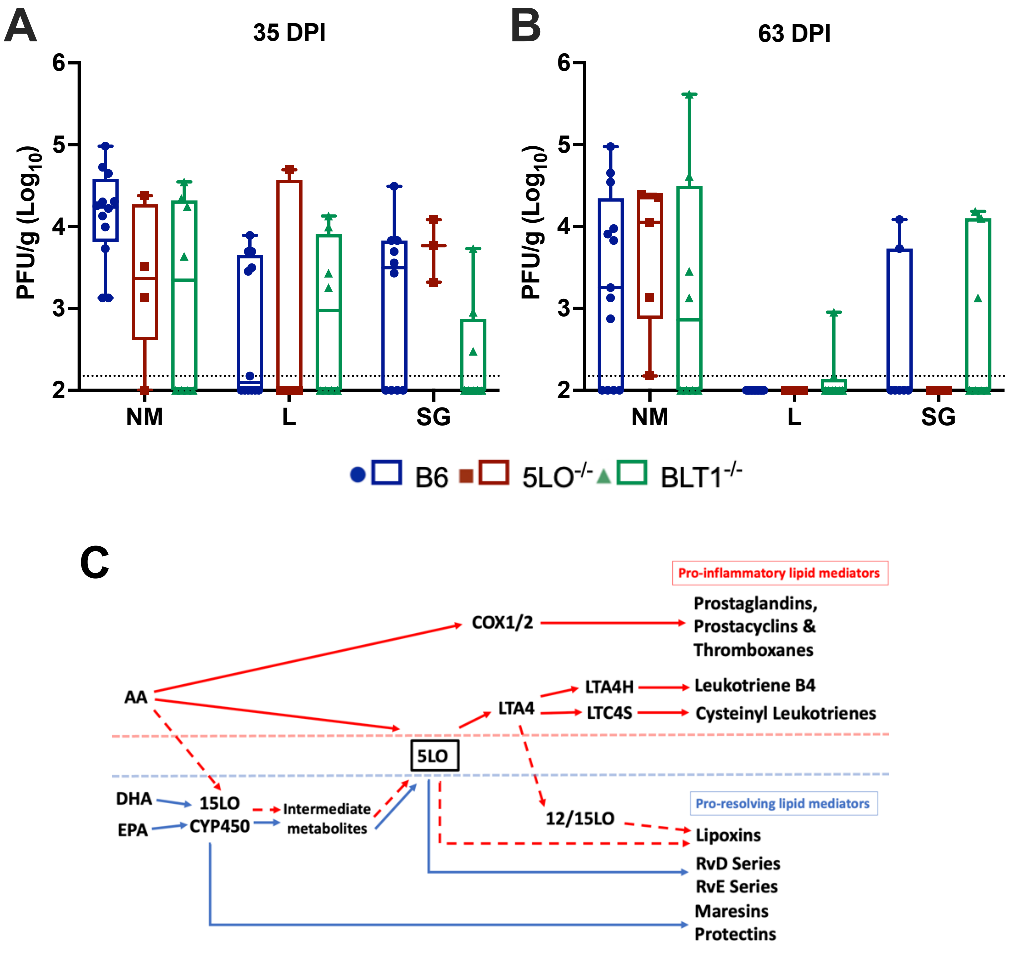

Supplementary Figure 1. LTB4 does not alter viral persistence in mucosal tissues. Plaque assay quantification of viral titers at (A) 35 DPI and (B) 63 DPI in the NM lungs, and SG of i.n. infected B6, $5LO^{-/-}$, and $BLT1^{-/-}$ mice. Box and whiskers plots graphed with min. to max., showing individual values of each mouse ranging from n=3-12 mice per group from at least two independent experiments. Dotted lines represent the assay limit of detection. (C) Simplified pathway of lipid mediator production derived from AA, DHA, and EPA. No significant differences were observed. (Forsythe and Welch One-way ANOVA of log transformed data: A-B) * = $p \leq 0.05$, ** = $p \leq 0.01$, *** = $p \leq 0.001$.

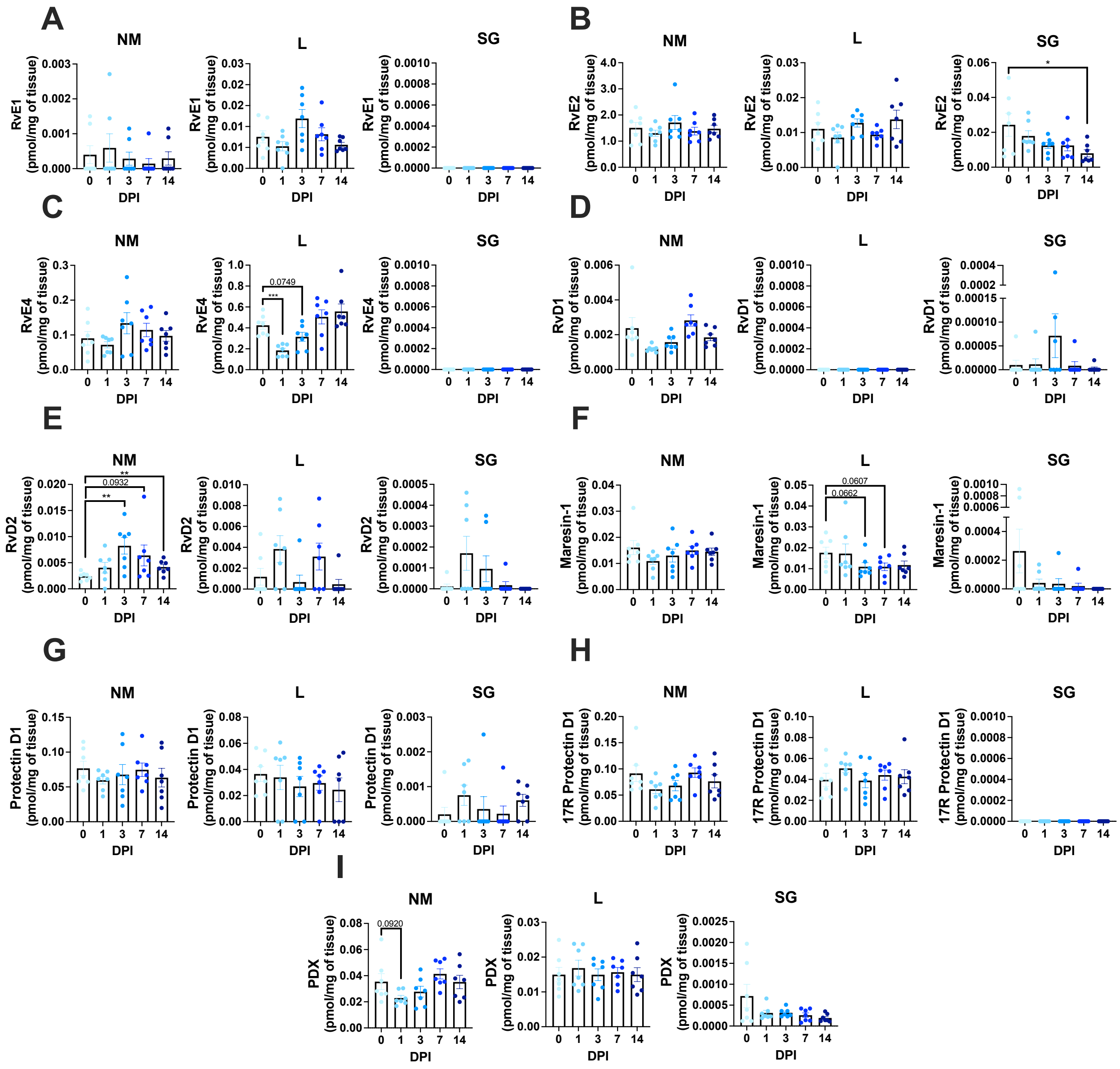

Supplementary Figure 2. DHA and EPA derived lipid mediators are produced at steady state and throughout CMV infection in the NM, lungs, and SG. Tandem mass spectrometry quantification of (A) RvE1, (B) RvE2, (C) RvE4, (D) RvD1, (E) RvD2, (F) Maresin-1, (G) Protectin D1, (H) 17R Protectin D1, and (I) PDX at 0, 1, 3, 7 and 14 DPI of the NM, lungs, and SG of i.n. infected B6 mice determined by MS-MS. Data were graphed as a scatter dot bar plot showing individual values of each mouse and mean with SEM. n=7 mice per time point. (Brown-Forsythe and Welch One-way ANOVA) * = $p \leq 0.05$, ** = $p \leq 0.01$, *** = $p \leq 0.001$.

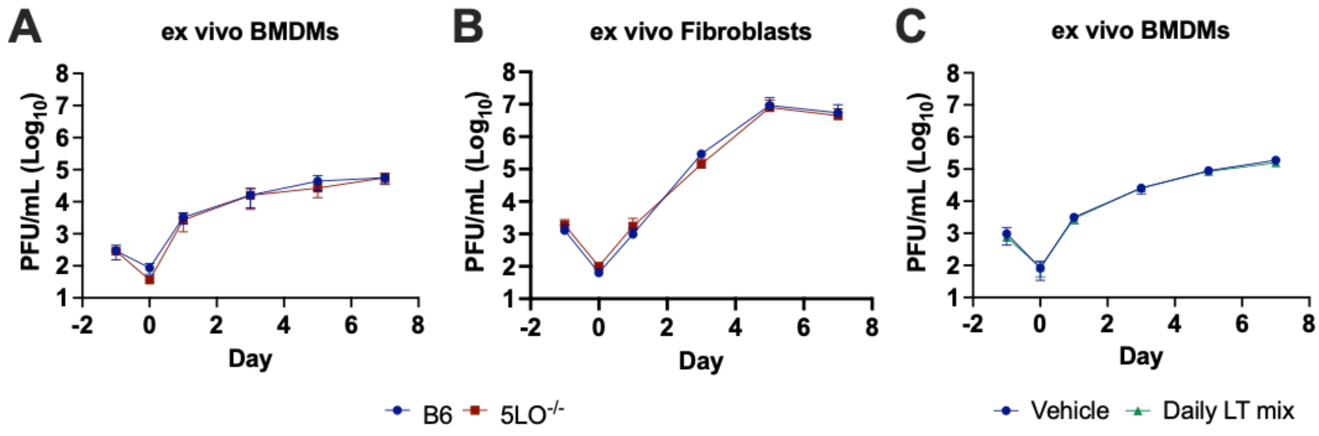

Supplementary Figure 3. Neither 5-lipoxygenase deficiency nor leukotriene treatment play a direct role in regulating MCMV growth. Multistep growth curves show viral growth in (A) BMDMs and (B) primary fibroblasts from B6 and $5LO^{-/-}$ mice or (C) BMDMs from B6 mice treated daily with vehicle or a leukotriene mixture (10 µM each of LTB4, LTC4, LTD4, LTE4, LTF4, and N-acetyl LTE). Fibroblasts and BMDMs were infected with K181-MCMV at an MOI of 0.1. Cells and supernatant were collected at input, 0, 1, 3, 5 and 7-days post infection and viral titers were measured by plaque assay on M2-10B4 cells. Data are shown as mean ± SEM from 2 individual experiments, each performed in duplicate. No significant differences were observed. (Two-way ANOVA) * = $p \leq 0.05$, ** = $p \leq 0.01$, *** = $p \leq 0.001$.

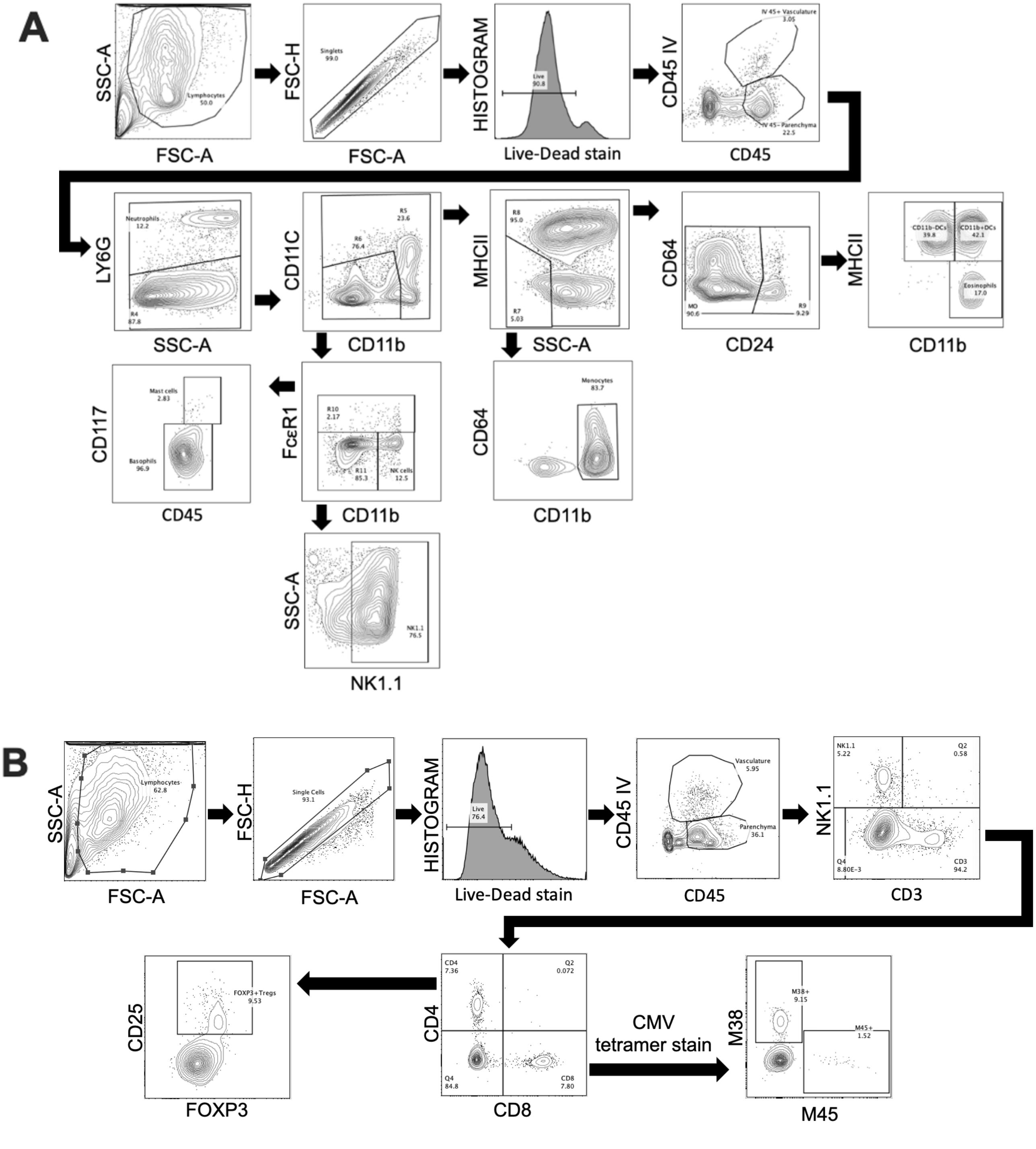

Supplementary Figure 4. Representative flow cytometry gating strategy. Data from B6 mice intranasally infected with 10^6 pfu K181-MCMV in 10 μL/naris. Shown is data from the NM and gating strategy for (A) myeloid cells and (B) T cells and antigen specific T cell responses.

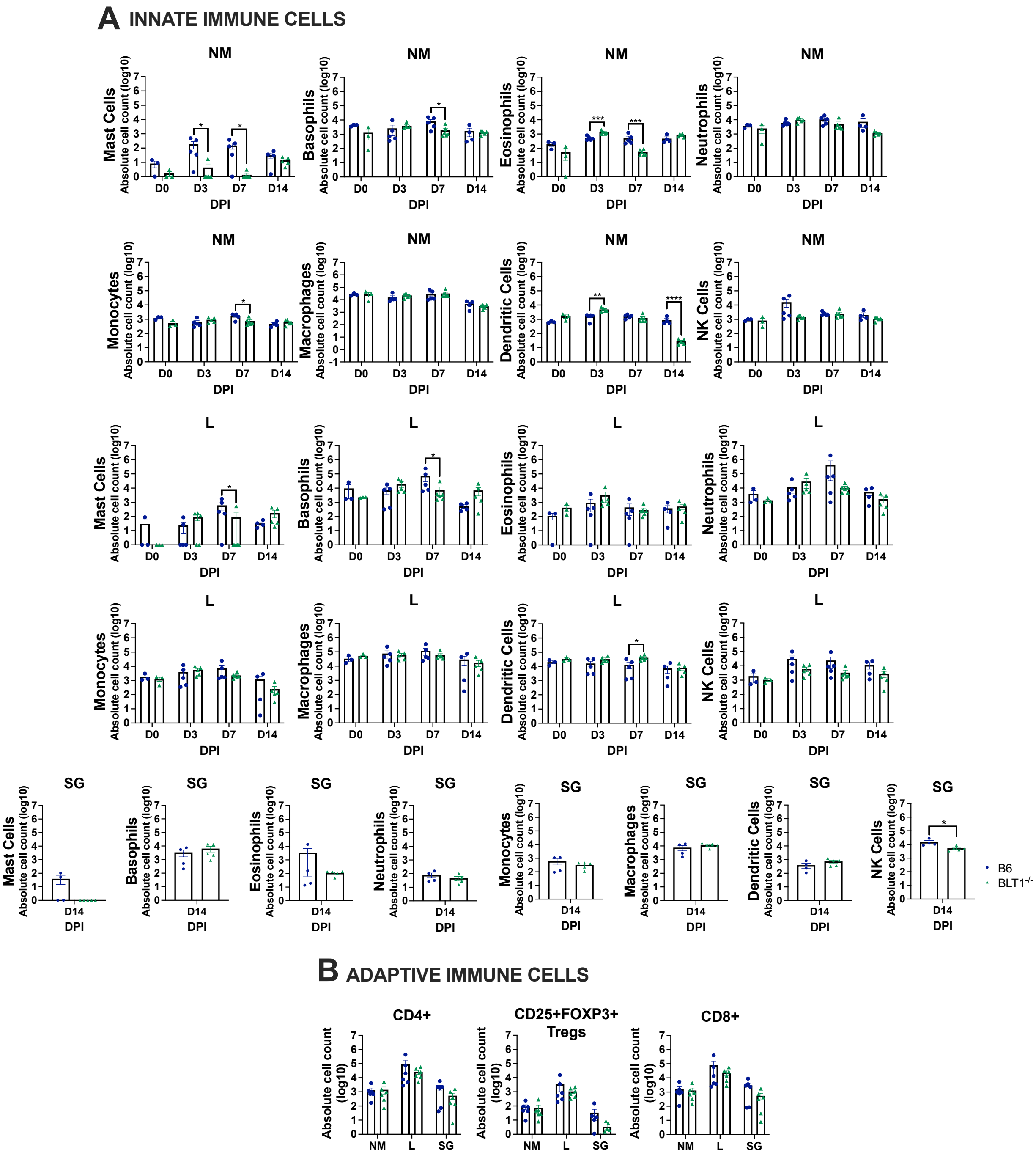

Supplementary Figure 5. Cellularity of the NM, L and SG is minimally impacted by BLT deficiency. Absolute cell counts of cells were determined by flow cytometry. Shown are (A) innate immune cells in the NM and lungs over time and SGs at 14 DPI and (B) T cell subsets at 14 DPI in the NM, lungs, and SG of i.n. infected B6 and $BLT1^{-/-}$ mice. Data are graphed as a scatter dot bar plot showing individual values of each mouse and mean with ±SEM. n=3-6 mice per group from at least two independent experiments. Asterisks represent significant differences in absolute cell count. (Welch's t test of log transformed data) * = $p \leq 0.05$, ** = $p \leq 0.01$, *** = $p \leq 0.001$.

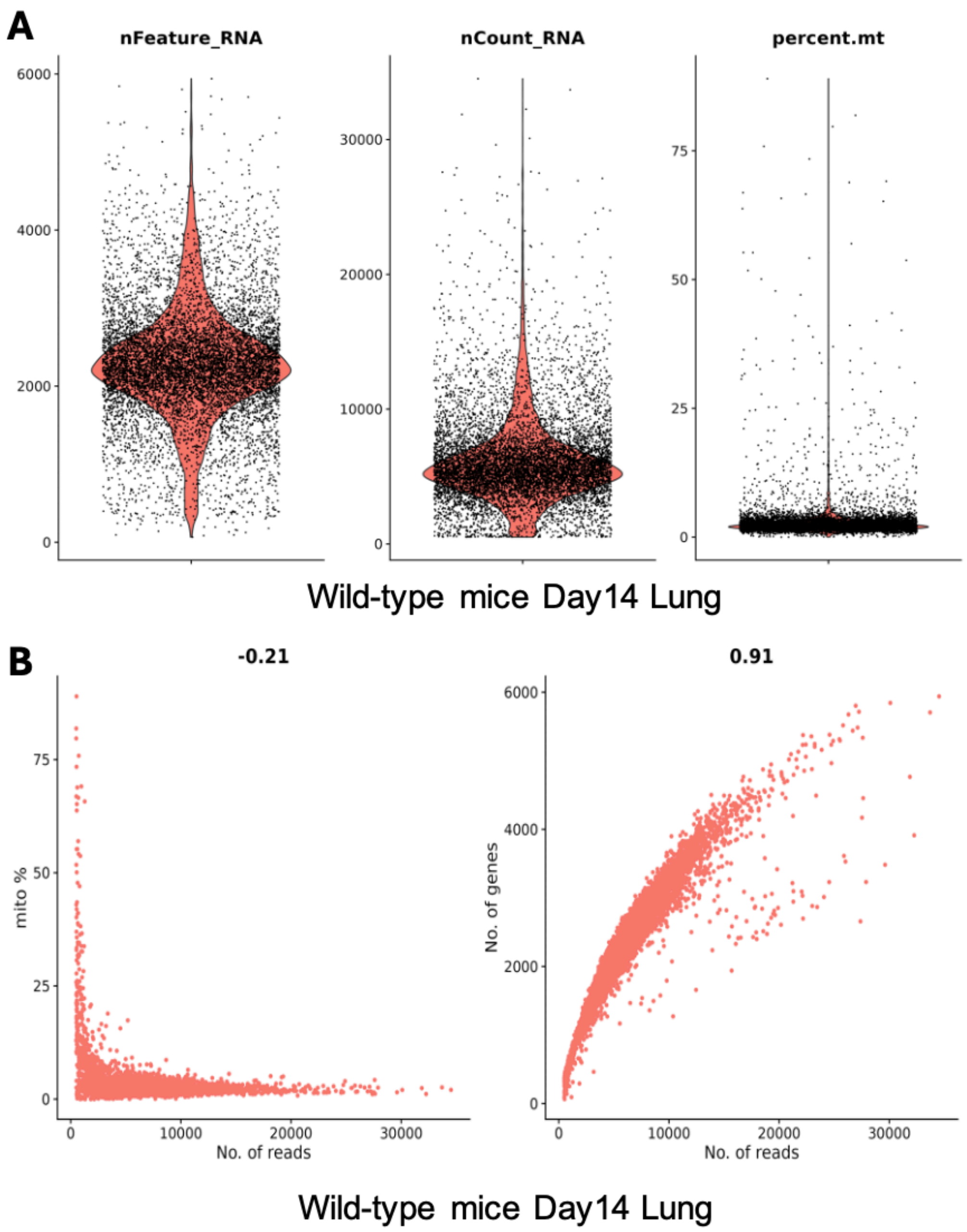

Supplementary Figure 6. Quality control of WT lung single cell data 14DPI. (A) The violin plots show the number of genes, unique molecular identifiers, and percentage of mitochondrial genes in each cell of the indicated sample. (B) The scatterplots show the relationship between the mRNA reads and the percentage of mitochondrial genes (left) or the number of genes (right).

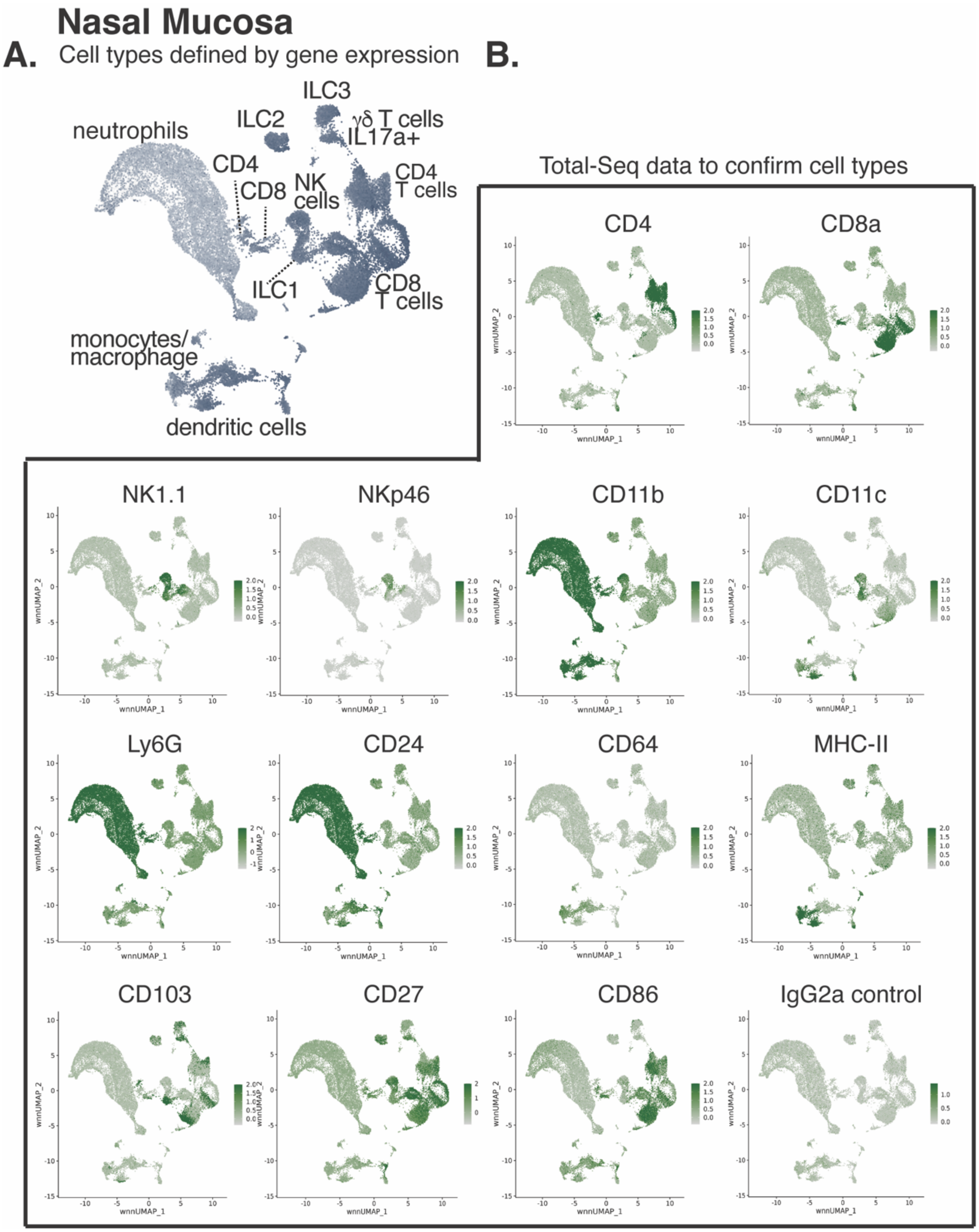

Supplemental Figure 7. CITE-seq of cells in the NM. To confirm cell types identified in the NM, we compared cells defined by gene expression profiles with reads from antibody-derived tags indicating protein levels of each marker. (A) Cells from the NM are labeled by cell types as defined by gene expression profiles. (B). Representative plots of the indicated antibody-derived tags.

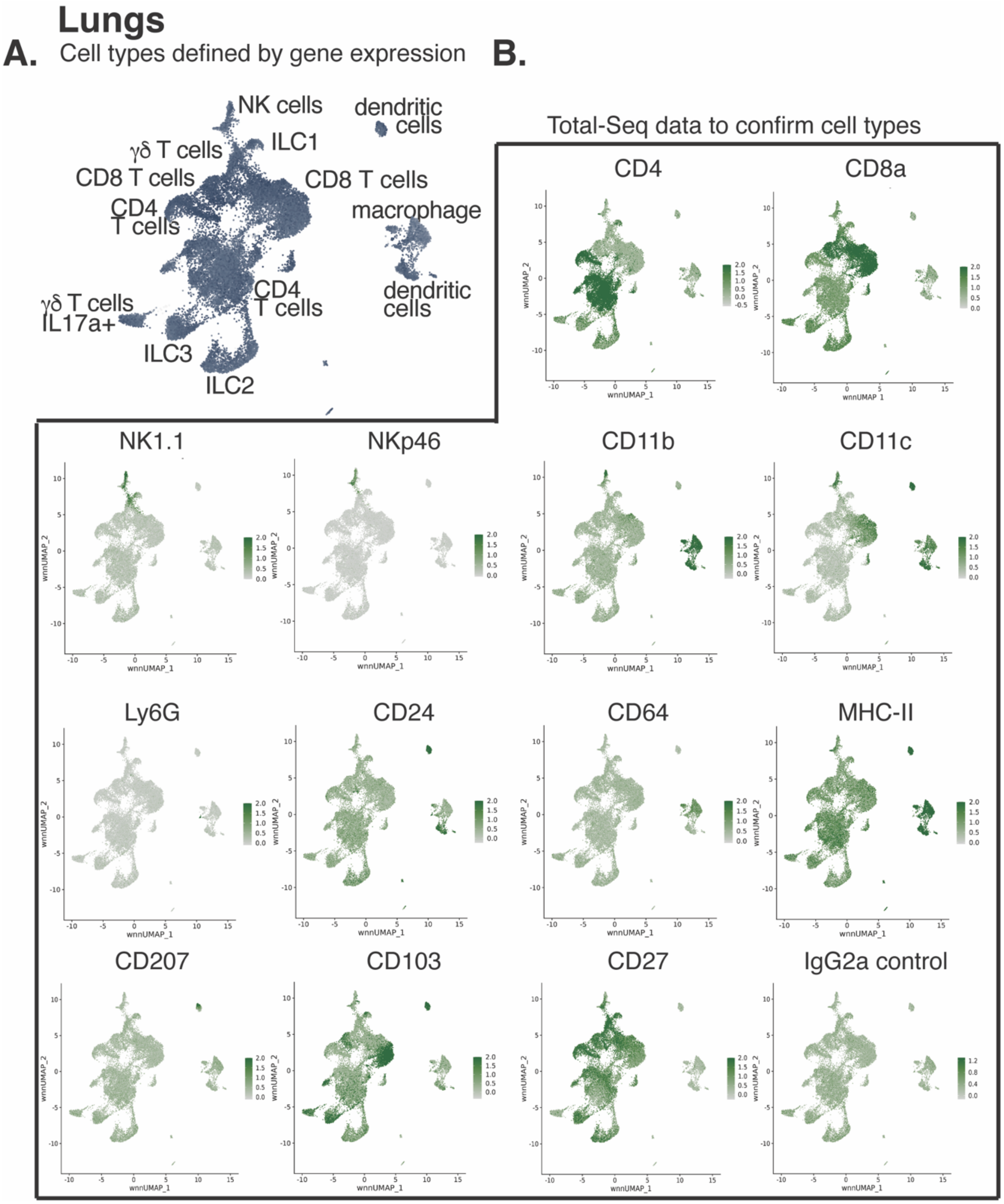

Supplemental Figure 8. CITE-seq of cells in the lungs. As in Supplemental Figure 6, we confirmed cell types in the lung by comparing cells defined by gene expression profiles with reads from antibody-derived tags indicating protein levels of each marker. (A) Cells from the lungs are labeled by cell types as defined by gene expression profiles. (B). Representative plots of the indicated antibody-derived tags.

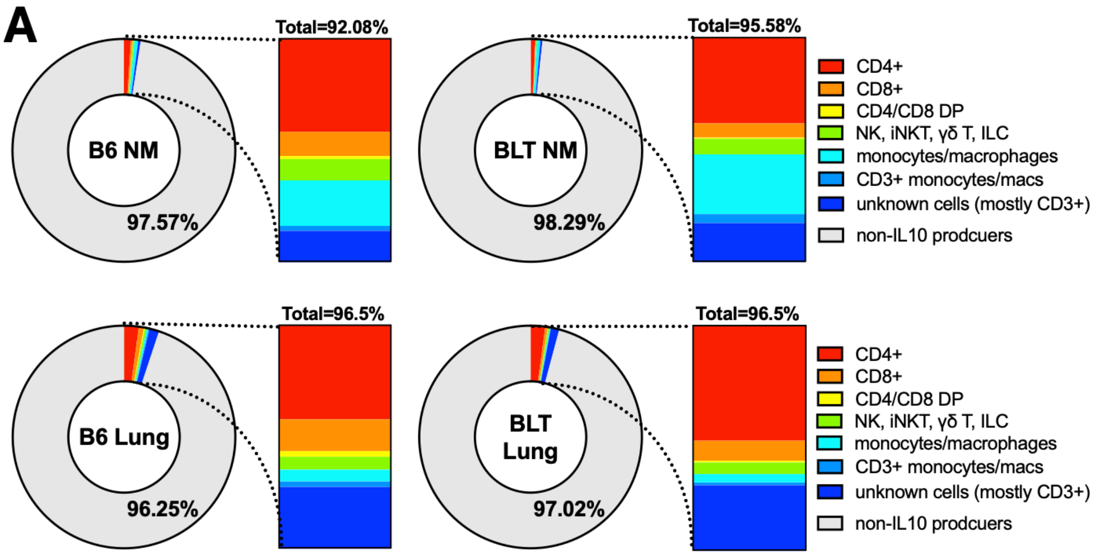

Supplementary Figure 9. Cell populations producing IL-10 are unchanged in BLT$^{-/-}$ mice. Cell types as defined in the methods and in the legend for Figure 4 were assessed for IL-10 expression. Quantification of the cell-types producing IL-10 in the NM and lungs is graphed to show the frequency of IL-10-producing cells (colored sections of the circles) among all cells in each tissue and within all IL-10-producers (stacked bar graphs). Numbers in the circles indicate the percentage of cells that did not express IL-10. Numbers on top of the stacked bar graph indicate the sum of all subsets as a percentage of all IL-10 producers. As in Figure 4, IL-10^{+} innate lymphocytes expressing either Zbtb16, Ncr1 or Trdc were pooled together (green). Here, monocytes and macrophages were divided into CD3^{+} and CD3^{-}. Again, an undefined population of cells was identified that produced IL-10 (darker blue). As described in Figure 4, these cells were Zbtb16^{-}, Ncr1^{-}, Trdc^{-}, CD14^{-}, Adgre1^{-}, Fcgr1^{-}, Fcgr3^{-}, CD4^{-}, CD8a^{-}, CD8b1^{-} and many of these undefined cells expressed CD3.

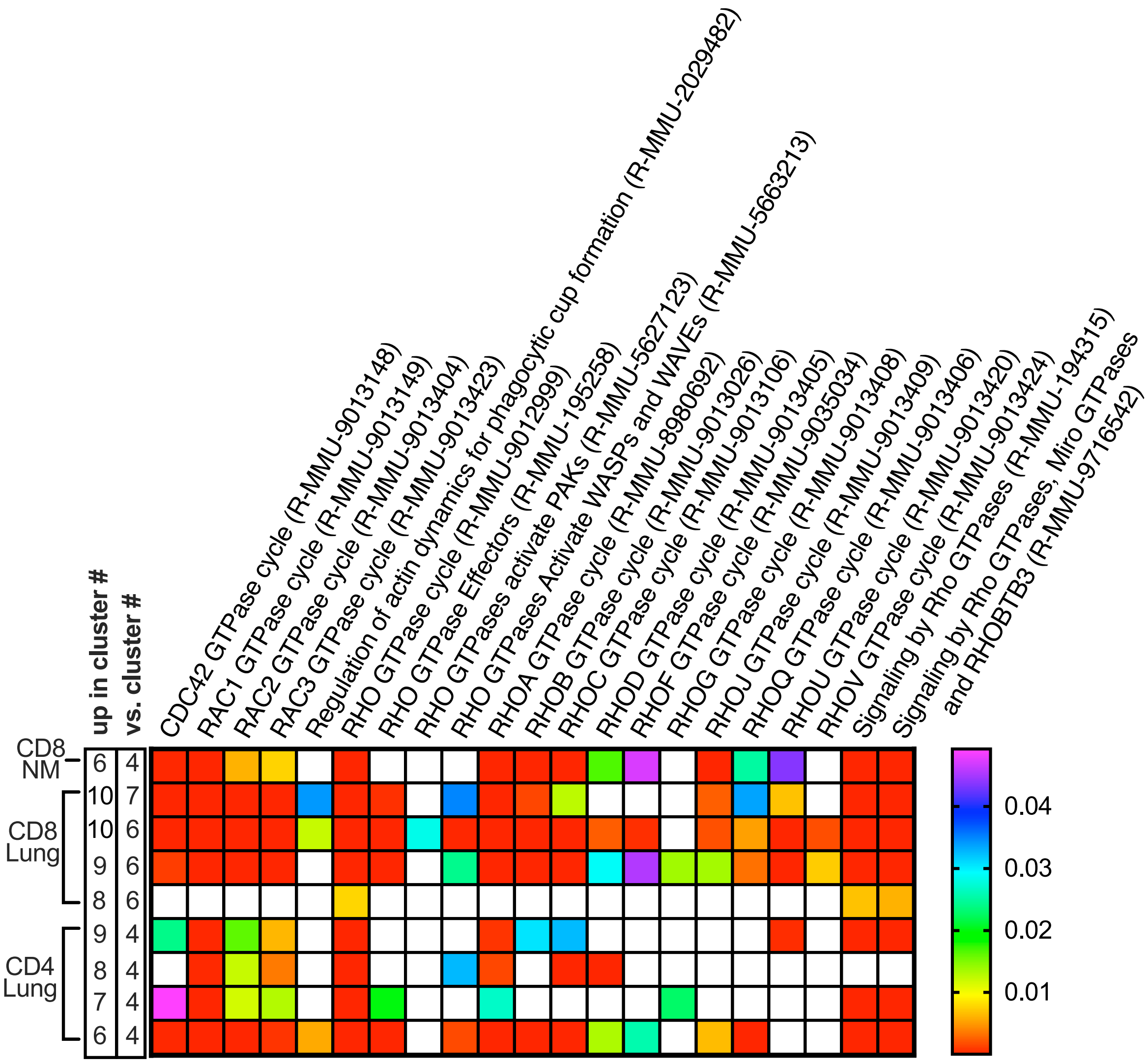

Supplementary Figure 10. Enrichment of Rho/Rac/cdc42 pathways in clusters lacking in BLT1$^{-/-}$ T cells. Clusters that were relatively enriched in B6 T cells were compared to clusters enriched in BLT$^{-/-}$ T cells (as in Fig 5C). Genes that were significantly upregulated (adjusted p-value <0.2) in the indicated B6 T cell enriched clusters vs. BLT$^{-/-}$ T cell enriched clusters were assessed for overrepresentation within Reactome pathways. The heat map shows the False Discovery Rate (FDR) for the indicated pathways identified as overrepresented among upregulated genes in B6 T cell enriched clusters. Unfilled boxes had no significant enrichment of the indicated pathways.

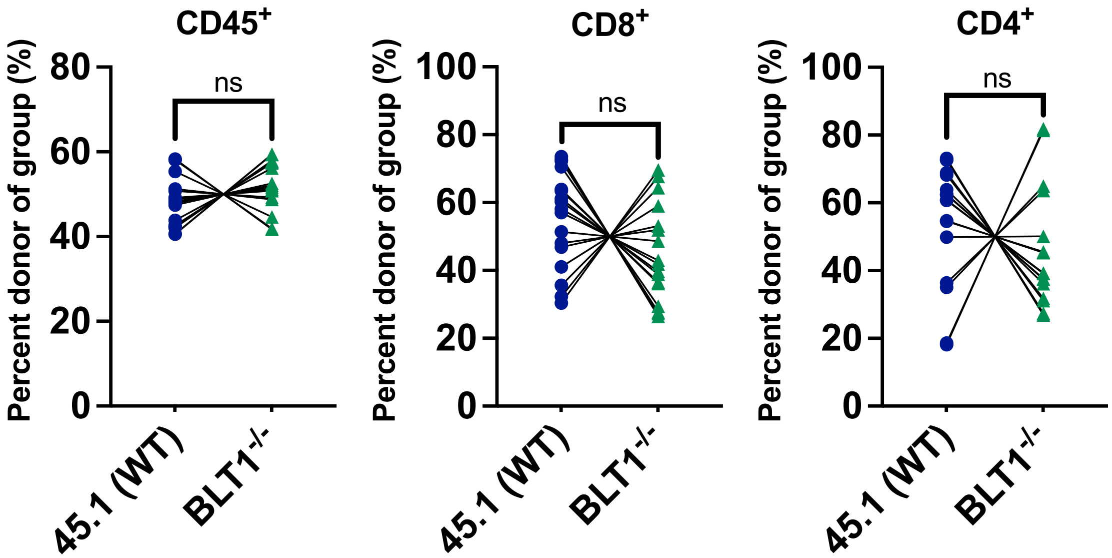

Supplemental Figure 11. Donor composition is comparable in the vasculature of mixed bone marrow chimera mice before infection. Irradiated recipient TCRβ$^{-/-}$ mice received BLT1$^{-/-}$ (CD45.2) and WT (CD45.1) bone marrow cells at a ratio of 2.5:1. Mice were then rested six-eight weeks and assessed for CD45^{+} cells, CD8^{+} and CD4^{+} T cells in the peripheral blood by flow cytometry. Data is representative of 17 mice per group from five independent experiments. No significant differences were observed as assessed by paired t test) * = $p \leq 0.05$, ** = $p \leq 0.01$, *** = $p \leq 0.001$

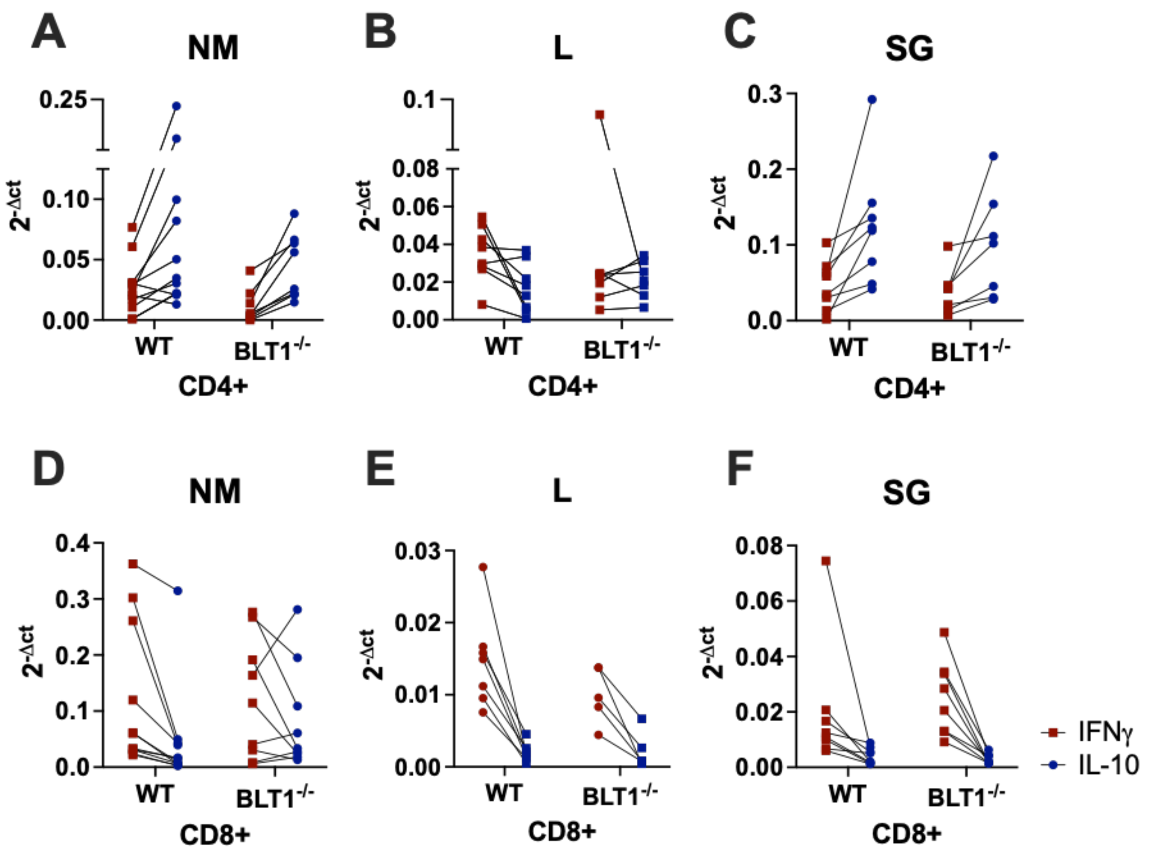

Supplementary Figure 12. BLT1$^{-/-}$ and WT T cells transcribe IFN-γ and IL-10 at similar levels. WT and BLT1$^{-/-}$ CD4^{+} and CD8^{+} T cells that localized to the tissue parenchyma were sorted from mixed bone marrow chimeras at 14 DPI. Shown are the levels of IFN-γ and IL-10 transcripts as assessed by qRT-PCR. (A-C) Quantity of IL-10 and IFN-γ transcripts in sorted WT and BLT1$^{-/-}$ CD4^{+} T cells from the indicated tissues. (D-F) Quantity of IL-10 and IFN-γ transcripts in sorted WT and BLT1$^{-/-}$ CD8^{+} T cells from the indicated tissues. Data is representative of 5-8 mice per group from at least two independent experiments. No significant differences were observed. (Wilcoxon test) * = $p \leq 0.05$, ** = $p \leq 0.01$, *** = $p \leq 0.001$.

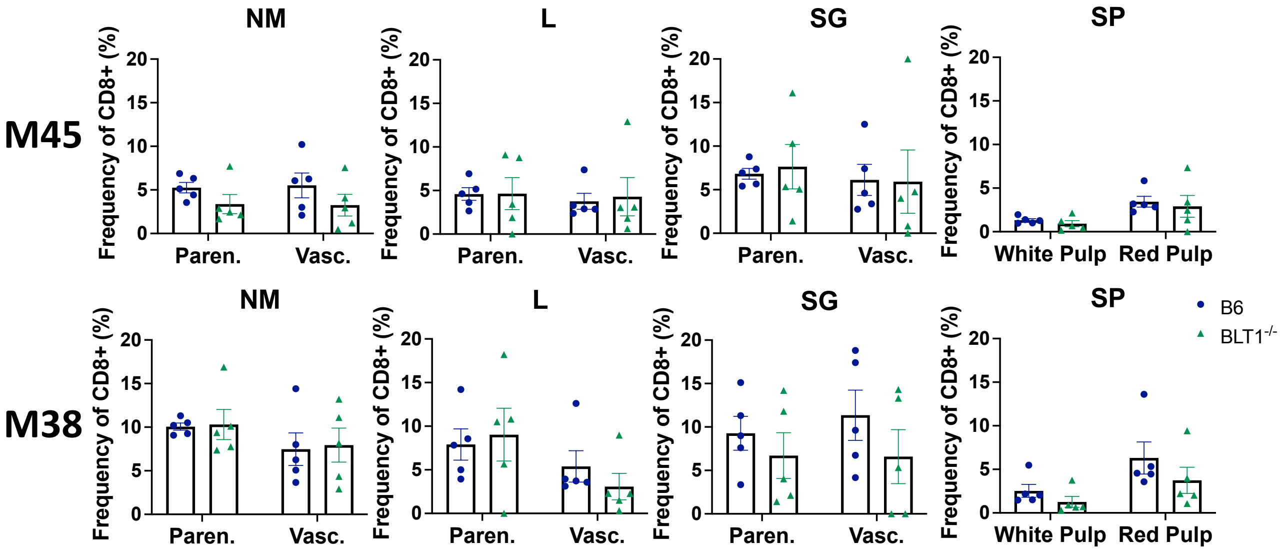

Supplemental Figure 13. BLT1$^{-/-}$ and WT CD8^{+} T cells mount similar antigen-specific responses at 10 DPI. Lymphocytes from NM, lungs, SG and SP from B6 and BLT1$^{-/-}$ mice at 10 DPI were analyzed by flow cytometry for tetramer-positive CD8^{+} T cell responses. Data are from 5 mice per group from at least two independent experiments. No significant differences were observed. (Mann-Whitney test) * = $p \leq 0.05$, ** = $p \leq 0.01$, *** = $p \leq 0.001$.

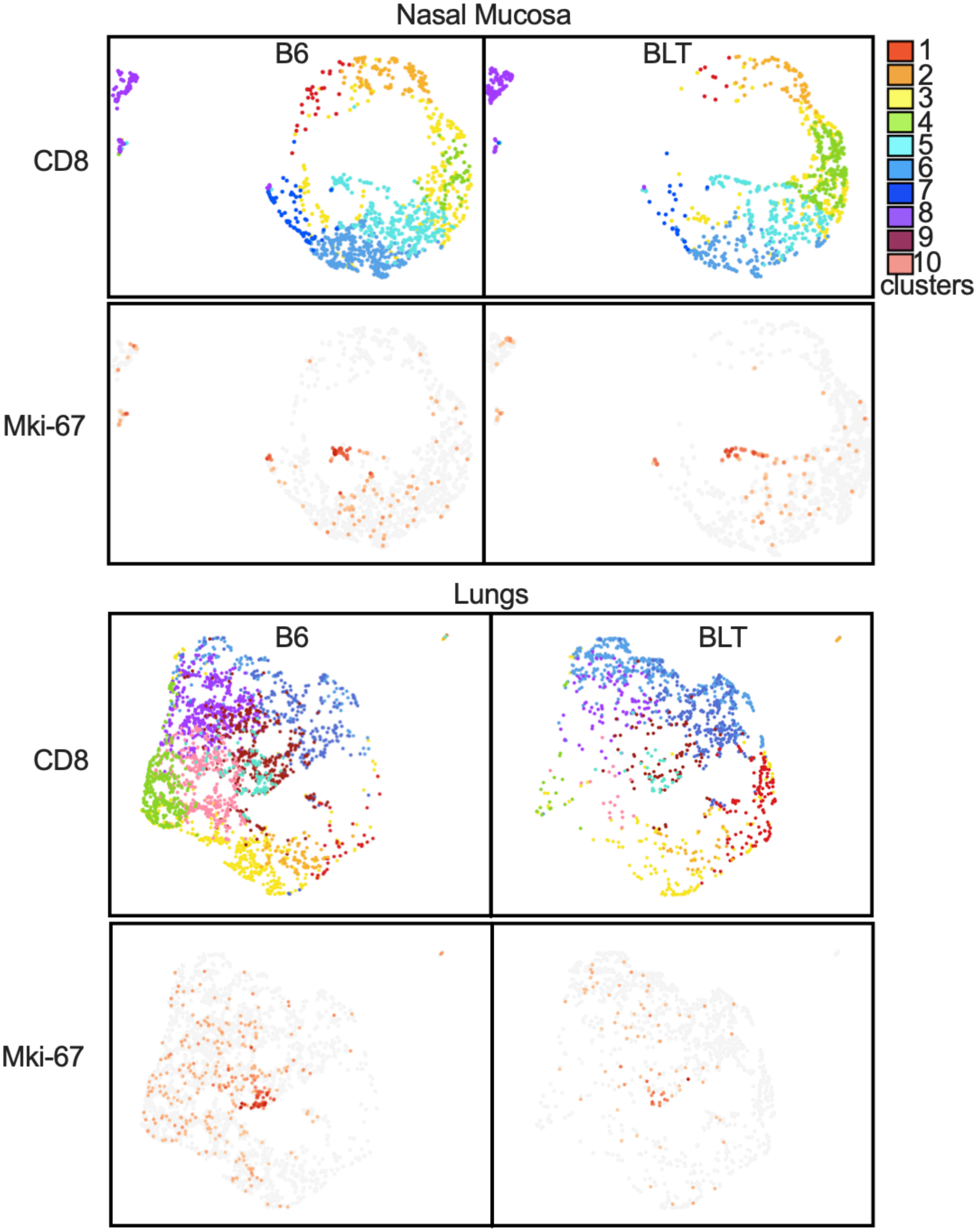

Supplementary Figure 14. BLT$^{-/-}$ CD8 T cells are not dividing as much as WT T cells. Clusters of $CD8^+$ T cells in the NM and lung were assessed for expression of the proliferation marker Ki-67. CD8+ T cells in both the NM and lungs of BTL1$^{-/-}$ mice show decreased expression of Ki-67 compared to cells of WT mice.
